# Surface plasmons interference nanogratings: wafer-scale laser direct structuring in seconds

**DOI:** 10.1038/s41377-022-00883-9

**Published:** 2022-06-23

**Authors:** Jiao Geng, Wei Yan, Liping Shi, Min Qiu

**Affiliations:** 1 Key Laboratory of 3D Micro/Nano Fabrication and Characterization of Zhejiang Province, School of Engineering, Westlake University, 18 Shilongshan Road, Hangzhou, 310024 Zhejiang Province China; 2 Institute of Advanced Technology, Westlake Institute for Advanced Study, 18 Shilongshan Road, Hangzhou, 310024 Zhejiang Province China

**Keywords:** Laser material processing, Nanophotonics and plasmonics

## Abstract

It is always a great challenge to bridge the nano- and macro-worlds in nanoscience, for instance, manufacturing uniform nanogratings on a whole wafer in seconds instead of hours even days. Here, we demonstrate a single-step while extremely high-throughput femtosecond laser scanning technique to obtain wafer-scale, highly regular nanogratings on semiconductor-on-metal thin films. Our technique takes advantage of long-range surface plasmons-laser interference, which is regulated by a self-initiated seed. By controlling the scanning speed, two types of nanogratings are readily manufactured, which are produced by either oxidation or ablation. We achieve a record manufacturing speed (>1 cm^2^ s^−1^), with tunable periodicity of *Λ* < 1 µm. The fractional variation of their periodicity is evaluated to be as low as ∆*Λ/Λ* ≈ 0.5%. Furthermore, by utilizing the semiconductor-on-metal film-endowed interference effects, an extremely high energy efficiency is achieved via suppressing light reflection during femtosecond laser nano-processing. As the fabricated nanogratings exhibit multi-functionality, we exemplify their practical applications in highly sensitive refractive index sensing, vivid structural colors, and durable superhydrophilicity.

## Introduction

Gratings have played an irreplaceable role in the progress of modern physics. In the early years, their development was primarily driven by spectroscopy. Nowadays, gratings are contributing to many areas including semiconductor manufacturing, metrology, space physics, nanotechnology, information technology, nanophotonics, etc^1–5^. Their manufacturing history can be traced back to 18th century^6^. But so far, it remains a big challenge to utilize a simple and low-cost method for high-speed manufacturing of large-area uniform nanogratings by the conventional nanofabrication techniques, including mechanical ruling^6^, focused ion beam milling (FIB)^7^, electron beam lithography (EBL)^8^, two-photon polymerization (TPP)^9^, laser direct writing (LDW)^10^, or thermal scanning-probe lithography (t-SPL)^11^. The manufacturing speeds of some other commercially available methods such as nanoimprinting^12^, photolithography^13^, or plasma beam etching^14^ are much faster, but they are multi-step and require masks, which are not inexpensive. In addition, the scanning beam interference lithography (IL) is another high-speed technique^13^, which is, however, lack of robustness. It requires extremely stable and complicated systems inside a specially designed enclosure. Any disturbances from heat plumes, noise, vibration, and others have to be banned^15^. Furthermore, the IL needs specific materials like light-sensitive organic photoresist.

Recently, femtosecond laser-induced periodic surface structures^16^, via ablation^17,18^, or oxidation/reduction^19–21^, has been considered as a low-cost, robust, single-step, maskless, flexible, and cheap candidate for producing large-area nanogratings. They originate from interference between the incident laser and surface electromagnetic waves (SEWs), especially the surface plasmon polaritons (SPPs), capable of forming nanogratings on a wide range of plasmonic materials, including metals, semiconductors, and semi-metals either when doped or under intense femtosecond laser irradiation^16,22^. From the practical standpoints, two important features are most concerned: the long-range uniformity and manufacturing speed. The nanogratings ablated by long-range SEWs are easily distorted by surface debris, residual heat and multiple random seeds, leading to poor uniformity. The oxidation-induced nanogratings, in terms of localized SEWs, are extremely uniform thanks to a nonlocal feedback mechanism and its robustness against surface debris^19,20^, but its manufacturing speed is very slow. In other words, the manufacturing speed and uniformity are in conflict with each other.

**Fig. 1:**
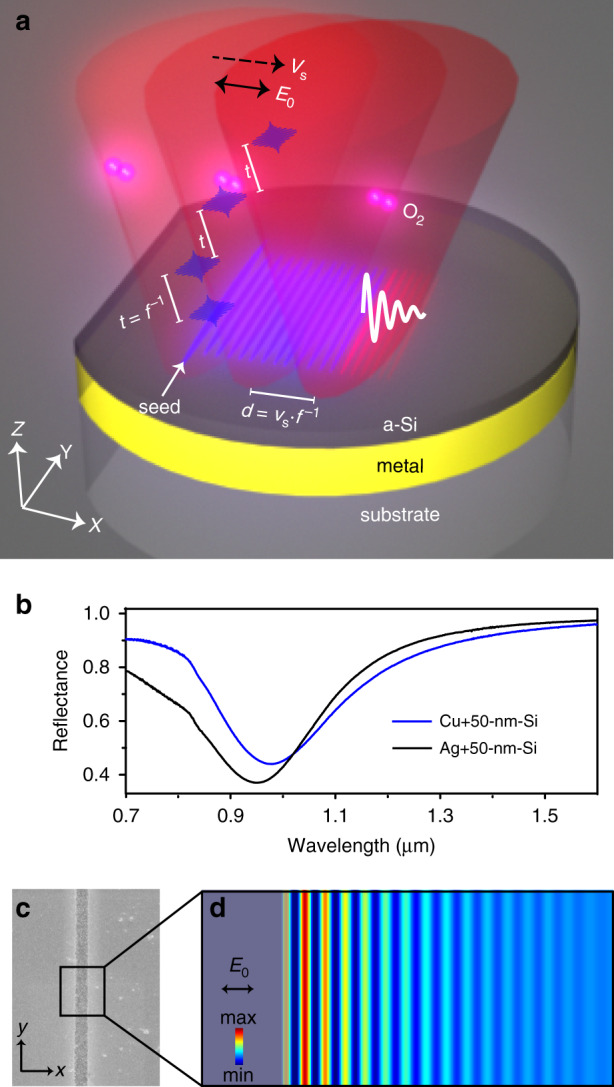
Scheme of femtosecond laser-induced self-organization of nanogratings on silicon-on-metal thin films. **a** A line-focused laser is employed to manufacture wafer-scale nanogratings on thin-film absorbers, consisting of an amorphous Si coating on a low-loss metal film such as silver or copper. When scanning the samples along *x*-direction (dashed arrow), parallel nanostripes are replicated from a seed. *f*: laser repetition rate; *t* = *f*^−1^: time interval between adjacent pulses; *v*_*s*_: scanning speed; *d* = *v*_*s*_*f*^−1^: spatial distance between adjacent pulses. *E*_0_: laser polarization direction. O_2_: oxygen, which plays an important role in the grating formation. **b** Normal incidence reflection spectra of 100-nm-thick Cu and Ag coated with 50 nm of Si. **c** SEM image of a single pulse-induced ablative slit on Si film. Its width is measured to be <2 µm. **d** Numerical illustration the formation of standing wave at air-Si interface via laser-SPP interference. In simulation, we set a 2-µm-width slit indentation (dark area) as seed in the Si film to excite the SPPs

We solve this contradiction by employing long-range SPPs that are excited by a single regular seed, along with preoxidation-assisted ablation. Femtosecond pulses are focused by a cylindrical lens onto silicon-on-metal (SOM) absorbers (Fig. 1a, b), forming a line-shaped beam profile. By carefully adjusting the peak laser fluence at the center of focal spot to reach ablation threshold of silicon, we obtain a high aspect ratio slit after irradiation of a single pulse (Fig. 1c). Its length in *y*-direction is *d*_*l*_ > 6 mm while its width in *x*-direction is only ∼2 µm. This slit acts as a seed, which launches SPPs, and leads to a parallel standing wave via laser-SPPs interference (Fig. 1d). The seed-initiated standing wave is robust against surface debris (Supplement Fig. S1). Therefore, nanograting with an orientation parallel to the seeding slit is formed in the constructive interference regions. The grating periodicity is fundamentally determined by the wavelength of scattered SPPs that propagate on SOM thin-film surfaces. It can be predicted from the dispersion equation,

1 $$1 + \frac{{\varepsilon _m\alpha _{air}}}{{\alpha _m}} = \tan \left( {\alpha _{Si}h} \right)\left[\frac{{\alpha _{Si}}}{{\alpha _{Si}\alpha _{air}}} - \frac{{\varepsilon _{Si}\alpha _m}}{{\varepsilon _m\alpha _{Si}}}\right]$$

here, $\varepsilon _{m,\,Si,\,{\rm{and}}\,{\rm{air}}}$ denote the relative permittivity of metal, Si, and air, respectively; $\alpha _{m,\;Si,\;air} \equiv \sqrt {\frac{{\omega ^2\varepsilon _{m,\;Si,\;air}}}{{c^2}} - k_{spp}^2}$ with wavenumber of SPPs *k*_*spp*_; *h* denotes the thickness of the Si film. Equation (1) suggests that SPPs wavelength relates to material parameters, film thickness, and also light wavelength. Among these factors, the most convenient way to control SPPs wavelength, i.e., nanograting periodicity, is simply to tune laser wavelength.

## Results

When scanning the samples along *x*-direction (Fig. 1a), the formation of nanogratings is similar to the toppling of dominoes, with existing nanogratings as seeds to guide the lateral generation of new parallel lines. As a result, there exist no phase shifts and deviations in the periodicity. Importantly, the long-range SPPs supported by the low-loss metals allow a large distance between adjacent pulses (*d*, Fig. 1a), and thus a high scanning speed (*v*_*s*_). Interestingly, the formation mechanisms of nanogratings are highly sensitive to the scanning speed (or total accumulated fluence: *F*_*t*_). At low scanning speed, the nanogratings are generated by oxidation. With high speed while at the same single pulse fluence, ablation process is dominant. In addition, the SOM samples are designed to be resonant with our laser at 1030 nm (Fig. 1b). Their antireflection improves the energy efficiency during laser processing, because for a given pulse energy, one could expand the beam spot to utilize a larger *d*_*l*_. A high *v*_*s*_ and a large *d*_*l*_ result in a fast-manufacturing speed *v*_*m*_ (cm^2^ s^−1^) = *v*_*s*_ × *d*_*l*_.

**Fig. 2:**
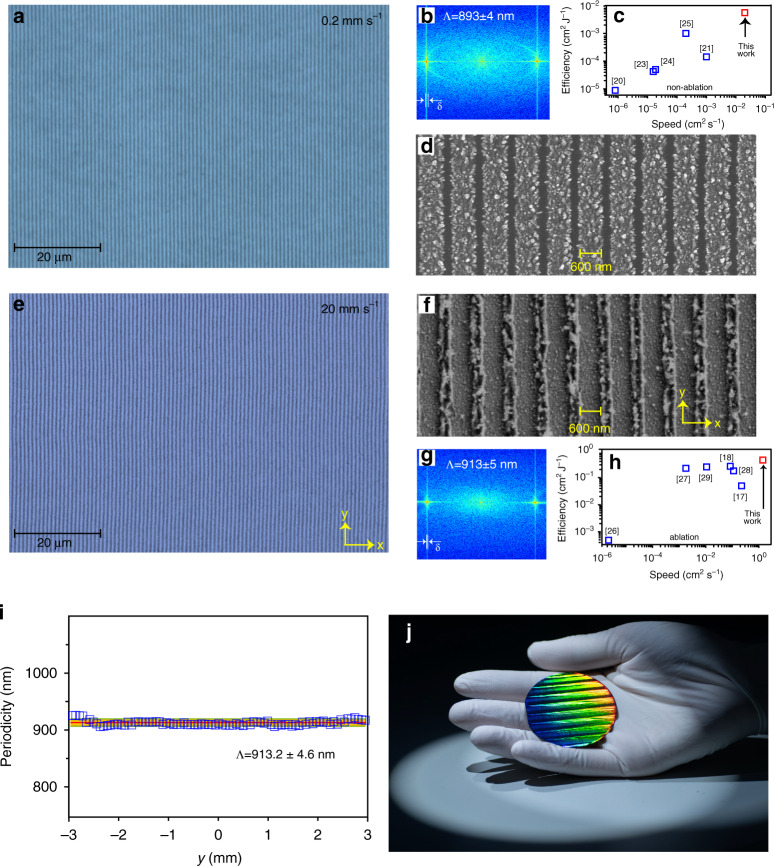
Characterization of femtosecond laser-written nanogratings. Microscopy images of nanogratings that are fabricated with a scanning speed of *v*_*s*_ = 0.2 mm s^−1^ (**a**) and *v*_*s*_ = 20 mm s^−1^ (**e**). Their 2D Fourier transforms and high-resolution images are displayed in **b**, **d** and **f**, **g**, respectively. *δ* denotes standard deviation of the periodicity. At low scanning speed, the nanogratings are self-organized via oxidation, while at high speed, they are produced through ablation. Comparison of manufacturing speeds and energy efficiency of our works with other high-speed manufacturing of nanogratings in terms of non-ablation processes (**c**) including oxidation in refs. ^20,23,24^, reduction in ref. ^21^, and phase change in ref. ^25^ or ablation (**h**). Please note that ref. ^29^ employed the scanning beam interference lithography. **i** Periodicity of ablative nanogratings along *y*-direction. Here *y* = 0 denotes the center of laser spot. The ablative trace along *y*-direction is 6 mm, shorter than the oxidized trace of 10 mm. **j** photograph of a 2-inch single-crystalline Si wafer coated with 100 nm of Ag and 50 nm of amorphous Si. The vivid rainbow color of the wafer indicates the existence of highly regular nanogratings. The wafer-scale nanogratings were produced within 20 s

Figure 2a shows a representative microscopy image of femtosecond laser-written nanogratings in the form of periodic oxidation. The incident single pulse fluence is 0.08 J cm^−2^. Its two-dimensional fast Fourier transform (2D-FFT, Fig. 2b) spectrum suggests that the period and its standard deviation (*δ*) is *Λ* ± *δ* = 893 ± 4 nm. In terms of energy-dispersive X-ray spectroscopy (EDX) and X-ray photoelectron spectroscopy (XPS), we confirm that the gratings are produced by periodic surface oxidation (Supplement Fig. S2). The orientation of the SPPs-induced nanogratings is perpendicular to the laser polarization. This differs from the traditional self-organized nanograting via oxidation^19,20^. The scanning speed and length of oxidation trace in *y*-axis are *v*_*s*_ = 200 µm s^−1^ and *d*_*l*_ = 1 cm, respectively. Therefore, compared with other high-throughput techniques also based on nonablative effects^20,21,23–25^, we achieve a highest energy efficiency (0.5 mm^2^ J^−1^), as well as a record manufacturing speed of *v*_*m*_ = 0.02 cm^2^ s^−1^ (Fig. 2c). It should be pointed out that our manufacturing speed is improved by several orders of magnitude with respect to the short-range SEWs-induced oxidation nanogratings^20,23,24^. The high-resolution SEM image (Fig. 2d) and its cross-sectional view (Supplement Fig. S3a) verify that the ridges of nanogratings are piled up by silicon oxide nanoparticles, with an average height of 50 nm (supplement Fig. S3b), and duty cycle of 67%. The formation of oxidation nanogratings on SOM films is rather flexible. For instance, they can be manufactured on non-planar surfaces (Supplement Fig. S4). Two-dimensional gratings can be produced via circularly polarized laser (Supplement Fig. S5). The resonant wavelength of the films and the periodicity can be tuned by varying the thickness of the Si coating layer (Supplement Fig. S6). When *v*_*s*_ spans from 500 µm s^−1^ to 2 mm s^−1^, the nanogratings present poor regularity (Supplement Fig. S7). However, once *v*_*s*_ exceeds 2 mm s^−1^, they resume excellent uniformity again.

Figure 2e displays the nanogratings that are written by *v*_*s*_ = 20 mm s^−1^ while at the same fluence of 0.08 J cm^−2^. Nevertheless, as indicated by the SEM image in Fig. 2f and confirmed by other evidence (Supplement Fig. S8), high *v*_*s*_-induced gratings are in the form of ablative grooves. They represent apparently different morphology from the low *v*_*s*_-induced oxidation gratings (Fig. 2d). Although the thickness of Si film is only 50 nm, the height of the grooves is measured to be >100 nm (Supplement Fig. S8). This is because the ablative nanoparticles redeposit a recast layer. At *v*_*s*_ = 20 mm s^−1^, the ablative trace in *y*-axis is measured to be *d*_*l*_ = 6 mm. In terms of the ablation effects, we achieve a record energy efficiency (0.4 cm^2^ J^−1^) and highest manufacturing speed of *v*_*m*_ = 1.2 cm^2^ s^−1 ^^17,18,26–29^. We successively evaluate the periodicity of the nanogratings by 2D-FFT along *y*-axis, that is, starting from one edge of the laser spot, crossing over the spot center and reaching the other edge of the laser spot. The periods of a total of 100 independent areas were measured, as shown in Fig. 2i. We confirm that its periodicity is highly uniform across the whole area, with a standard deviation of ∆*Λ* = 4.6 nm and thus ∆*Λ/Λ* ~ 0.5%. The typical tolerable error of groove spacing (*δ*) is directly related to its periodicity (*δ* < *Λ*/10 m)^6^, where *m* is an integer denoting the diffraction order. For the first-order diffraction in our case, the tolerance is *δ* = 90 nm. Therefore, the error of our nanograting satisfies the tolerance value. The insensitivity of the periodicity to laser intensity provides an important advantage of this method to create wafer-scale highly uniform nanogratings (Fig. 2j).

## Discussion

**Fig. 3:**
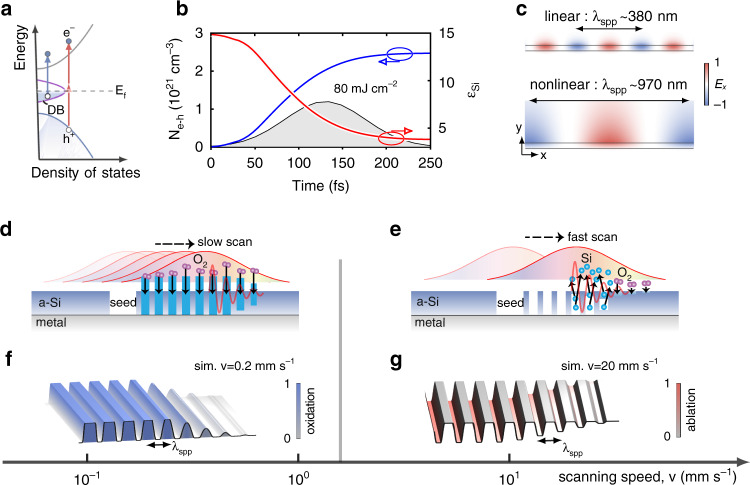
Formation dynamics of nanogratings. **a**–**c** Nonlinear modulations of SPPs by excitation of e–h pairs in Si-on-Ag films under intense ultrafast laser illumination. **a** Sketch of band structure of amorphous Si, and generation of e–h pairs by two-photon absorption as well as linear absorption due to dangling bonds with light energy lower than band gap. **b** Transient evolution of spatially averaged e–h pair density, *N*_e-h_, and the permittivity (real part) of amorphous Si, *ε*_si_, considering a Si-on-Ag film illuminated by a femtosecond laser pulse (shaded area). **c** Modal profiles of SPPs supporting by the Si-on-Ag film in the initial linear regime (upper panel) and with *ε*_si_ corrected by the e–h pairs (bottom panel). The thickness of the Si film is 50 nm, and the laser pulse has 130-fs duration, 0.08 J cm^−2^ fluence, and 1030 nm wavelength. **d**–**g** Sketch of forming oxidized-ridge (**d**) and ablative-groove (**e**) nanogratings by controlling laser scanning speeds, and their exemplified numerical simulations (**f**, **g**). Both experimentally and numerically, we observe the formation of oxidized-ridge gratings with a scanning speed lower than 0.5 mm s^−1^, and of ablative-groove gratings with a scanning speed higher than 2 mm s^−1^

To get a deep insight into the laser-SPPs interference-caused grating periodicity [cf. Eq. (1)], one has to consider the nonlinear modification of material parameters by femtosecond pulses. We identify that the permittivity change associated with electron-hole (e–h) formations in Si is dominant over other nonlinear effects, e.g., the Kerr nonlinearities. A comparative study of various nonlinear effects is given in Supplement 2.1 and Figs. S10–S11. For crystalline Si, the band gap is 1.1 eV, electrons are mainly ionized through interband linear absorption and two-photon absorption (TPA)^30^. However, for amorphous Si in our experiments, the dangling bonds may further enhance the linear absorption^31^. The dangling bonds enable optical absorption even when photon energy is below band gap (Fig. 3a)^32^. Under our experimental conditions, a single pulse can efficiently generate a high density of e–h pairs above 10^21^ cm^−3^, as numerically illustrated in Fig. 3b for a Si-on-Ag thin film. The details of nonlinear simulations are summarized in Supplement 2.3. Besides promoting material modifications needed for grating formations, such dense e–h pairs also significantly lower the permittivity of Si (red curve in Fig. 3b). With the nonlinearity-corrected material permittivity, the SPPs wavelength (about 970 nm) is found to agree reasonably with the grating periodicity observed in the experiments, as shown in Fig. 3c and Supplementary Fig. S11.

The formation of oxidized-ridge or ablative-groove nanogratings and its direct dependence on laser scanning speed are essentially a multi-pulse nonlinear phenomenon, wherein inter-pulse feedback is decisive. In case of slow scan (Fig. 3d), a fixed location on the surface is initially exposed to low fluence from the tail of the laser beam by many predecessor pulses, leading to adequate oxidization. Later, as the beam center arrives, the previously formed oxidized layer prevents the ablation even under high fluence, and an oxidized grating is thus formed. Experimentally, we observe that the formation of such regular oxidized gratings requires *v*_*s*_ < 0.5 mm s^−1^. This suggests that the oxidation process takes several hundreds of effective pulse number (*N*_*eff*_ = *fd*_*x*_*/v*_*s*_ > 800), corresponding to a total accumulated fluence *F*_*t*_ = *F* × *N*_*eff*_ > 64 mJ cm^−2^. In the opposite case of fast scan while at the same single pulse fluence (*F*_*t*_ < 16 mJ cm^−2^), the beam center rapidly passes across the shallow oxidized grating where the degree of oxidation is low (Fig. 3e). As a result, the formed incomplete-oxidation nanograting is removed and finally forms an ablative nanogratings. To verify the above physical processes, we simulate the formation of nanogratings under different scanning speeds (see Supplement 3 for simulation details). The oxidation protection against ablation is taken into account by setting the ablation threshold of SiO_2_ to be 2 mJ cm^−2^, that is about 10 times larger than the threshold of Si^33,34^. Figure 3f, g and Supplement Fig. S13 show the formations of the uniform oxidized-ridge and ablative-ridge gratings under the scanning speeds *v*_*s*_ = 0.2 and 20 mm s^−1^, respectively, which agree qualitatively with the experimental observations in Fig. 2d and f.

**Fig. 4:**
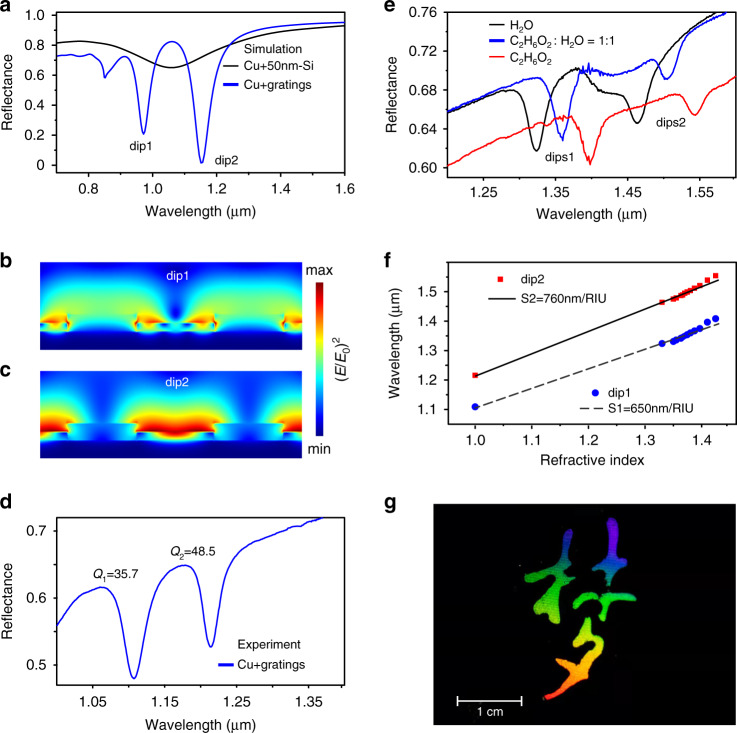
Applications of wafer-scale nanogratings in refractive index sensing and structural coloring. In applications, copper is used instead of silver because of its lower cost. **a** Numerically simulated reflection spectrum of a pristine thin-film absorber consisting of 50-nm-thick Si on 100-nm-thick Cu film, and reflectance of a one-dimensional thin-film grating. The grating periodicity is set to 900 nm, including 450-nm-width of Si and 450-nm-width SiO_2_. **b**, **c** The cross-sectional electric field distribution at the two observed dips. Experimentally measured reflection spectra of a laser-produced nanograting on Cu film in air (**d**) and in liquid environments (**e**). **f** Measured spectra dips versus refractive index of the involved liquids. **g** A photograph showing a colorful artistic word meaning “dream” that is written by femtosecond laser on SOM thin films

As the thin film nanogratings significantly alter the optical and mechanical properties of the SOM absorber, they exhibit multi-functional applications. For instance, as shown in Fig. 4a, the oxidation-induced nanogratings split the broadband resonant spectrum of SOM (black curve) into two narrowband remarkable dips (blue curve). Figure 4b, c shows the electric near-field distribution at these two resonant frequencies. The two reflectance dips correspond to near-field enhancement at the oxidized area (Fig. 4b), and at the pristine Si stripes (Fig. 4c), respectively. Figure 4d plots the experimentally measured reflection spectrum in air of the laser-written oxidation nanograting at *v*_*s*_ = 200 µm s^−1^. Indeed, two narrowband resonances are observed. Their quality factors (Q-factor) are evaluated to be up to *Q*_1_ = 35.7 and *Q*_2_ = 48.5, respectively. Optically resonant periodic surface nanostructures usually hold potential applications in refractive index sensing. To this end, narrow line width and hence steep slopes are desirable to track even small changes^35^. The localized surface plasmonic resonances are usually rather broad due to radiative and ohmic losses. An already exploited solution is to employ dark or subradiant modes to obtain narrow resonant spectra^36^. However, they are generally fabricated by electron beam lithography or focused ion beam, limiting their widespread and practical applications. Our wafer-scale, high Q-factor nanogratings provide a promising alternative. Figure 4e plots the measured reflectance in several exemplified liquids. We find that the reflectance dips are highly sensitive to the density of the ethylene glycol (C_2_H_6_O_2_). The dependence of resonant dips on the refractive index is depicted in Fig. 4f, showing an excellent linear relationship. The sensitivities (*S* = ∆*λ/*∆*n*) of these two dips are fitted to be *S*_1_ = 650 and *S*_2_ = 760 nm/RIU. The corresponding figure of merit (FOM) values are evaluated to be FOM_1_ = S_1_/FWHM = 20.9 and FOM_2_ = 30.4, where FWHM denotes full width at half maximum of the resonant spectra. The FOM values of our femtosecond laser-written macroscale nanograting are comparable to that produced by electron beam lithography^37^, rendering them more practical for biosensing.

Laser marking has become a popular method for industrial product identification^38^. Nevertheless, it traditionally produces contrast patterns which are monochromatic^39^. The self-organization of nanogratings by a single-beam femtosecond laser provides a versatile method for laser colorful marking, as it gives rise to viewing angle-dependent structural colors. The previous works have demonstrated that a low-oxygen ambience is desired for improving the brightness of structural colors on Si^39^, because the oxygen reduces the regularity of nanogratings. However, we find that the oxidation-induced highly regular nanogratings exhibit a very bright colors covering the entire visible spectrum from purple to red (Fig. 4g). The tunable color of the thin-film absorbers (Supplement Fig. S6), combining with the uniform grating-caused iridescence render them as attractive candidates for visual arts. In addition, the nanogratings also exhibit durable superhydrophilicity (Supplement Fig. S9), which hold potential in water harvesting, microfluidics, and self-cleaning applications.

In summary, we have demonstrated the high-speed manufacturing of wafer-scale, highly regular plasmonic nanogratings on SOM thin-film optical absorbers^40^ by femtosecond laser-induced self-organization. Such a two-layer SOM system solves several challenges in the research direction of laser nanofabrication, providing a flexible nanofabrication method for a variety of industrial applications^41^. For example, we have shown that the vivid structural colors arose from the gratings-induced diffraction can be employed for laser colorful marking and labeling, or even the visual arts. We also have verified that the produced dielectric gratings on metals support remarkable narrowband resonances, which provide excellent chips for biosensing. In addition to these, it can also be applied in other areas like thin-film optical filters, solar cells, photodetectors, nonlinear nanophotonics, etc. Further improvement of this method can be conducted from the following directions. First, one can further improve the scanning speed by using higher repetition-rate lasers and increase the beam diameter by using higher energetic like mJ-level lasers. Second, instead of Si, some wear-resistant absorbing materials, such as titanium aluminum nitride (TiAlN), can be used as the thin dielectric coatings to enhance the stability and lifetime of the samples^42^. Third, instead of the two-layer broadband absorber, multi-layer Fano-resonant thin-film absorbers^43^ will be of interest for producing nanogratings with this method.

## Materials and methods

### Experimental setup

We carried out the experiments with a diode-pumped ultrafast fiber amplifier system (Amplitude) delivering a repetition rate of *f* = 20 kHz, central wavelength of 1030 nm, pulse duration of 130 fs, beam diameter of 3 mm (at e^−2^). An optical parametric amplifier system was employed to tune the laser wavelength. The laser beam diameter was expanded to 12 mm by a telescope and then focused onto the target samples via a cylindrical lens with focal length of 75 mm. The cylindrical focusing formed a *d*_*y*_ = 12 mm long and *d*_*x*_ = 20 µm wide line spot on the surface of sample, giving rise to a high aspect ratio of *d*_*y*_*/d*_*x*_ = 600 elliptical focus. The delivered pulse energy at the sample was 160 µJ, corresponding to an averaged fluence of *F* = 0.08 J cm^−2^. An industrial camera was utilized for real-time monitoring of the surface structural colors during the processing. A half-wave plate combined with a linear polarizer was employed to control the pulse energies. The sample was installed on a three-dimensional translation stage. The scanning direction was along the laser polarization direction. It should be pointed that the maximum scanning speed of our translation stage is 20 mm s^−1^, which limits the ultimate manufacturing speed.

### Numerical simulation

We performed the numerical computations by using finite-difference-time-domain (FDTD) method (Lumerical FDTD solutions software package). The electric field distribution at Si-air interface and reflection spectra when existing periodic oxidized nanogratings on the thin-film absorbers were investigated. In Fig. 1d, a rectangular box with dimensions of 20 µm × 2 µm was set as the simulation region. The top and bottom boundaries were perfectly matched layers (PML), while periodic boundary conditions were applied in *x–y* plane. The light source was a *x*-polarized planar wave, which was normally incident onto the Si film from air. The slit was set to a width of 2 µm and depth of 50 nm, that is, the thickness of the Si film. The surface debris in Supplementary Fig. S1 was assumed to be SiO_2_, which came from the ablative slit and redeposited on Si film. Their size was 0.2 × 0.4 × 0.1 µm^3^. In Fig. 4a, the simulation region was 0.85 µm × 2 µm, with PML in *z*-axis and periodic boundaries in *x*–*y* plane. The width of the SiO_2_ stripe was 400 nm and height was 100 nm. Half of the stripe was immersed into the Si film. The light source was a *x*-polarized plane wave spanning from 400 nm to 2 µm. A monitor was set above the source to acquire the reflection spectra (Fig. 4a). The cross-sectional view electric field distribution in Fig. 4b, c was obtained by Fourier transformation of the time-domain electric field at a wavelength of 1030 nm.

### Sample fabrication

The thin films were evaporated with a magnetron sputtering system at room temperature (ULVAC CS200Z). The substrates were 2-inch single-crystalline silicon wafer. The silver films were coated via DC sputtering at power of 100 W, pressure of 0.3 Pa, flow rate of Ar of 150 sccm from the silver target located at distance 130 mm with the deposition rate 15 nm min^−1^. The copper films were coated via DC sputtering at power of 600 W, pressure of 0.3 Pa, flow rate of Ar of 100 sccm from the silver target located at distance 130 mm with the deposition rate 48 nm min^−1^. The Si films were coated via RF sputtering at power of 300 W, pressure of 0.3 Pa, flow rate of Ar of 70 sccm from the silicon target located at distance 90 mm with the deposition rate 6 nm min^−1^.

### Sample characterization

The scanning electron images, and energy-dispersive X-ray spectroscopy were performed by a field-emission scanning electron microscope (Carl Zeiss, Gemini450). The height of the nanogratings was measured by an atomic force microscope (Bruker, Dimension ICON). The surface and depth profile analysis of chemical components were performed by an X-ray photoelectron spectroscopy (Thermo Fisher Scientific). The milling and in situ imaging of the cross-sectional view of the nanogratings were carried out by a focused ion beam (Carl Zeiss, Orion Nano Fab). The Ga^+^ beam was used to mill the sample and He^+^ beam was for imaging.

### Linear reflection spectra, refractive index sensing, and superhydrophilicity

The variation of refractive index was controlled by precisely mixing ethylene glycol with pure water. The reflection spectra were measured by a Shimadzu UV-VIS-IR spectrophotometer (UV3600Plus+UV2700). The contact angle of the water droplet was measured by a contact angle goniometer (DATA PHYSICS OCA25, Germany).

## Supplementary information

Supplementary Information
