# Supplementary material for: Surface plasmons interference nanogratings: wafer-scale laser direct structuring in seconds
Source: Light Sci Appl. 2022 Jun 23;11:189. doi: 10.1038/s41377-022-00883-9 (PMC9226179; doi:10.1038/s41377-022-00883-9)
Supplement: Supplementary file 1 — Supplementary Information [file 41377_2022_883_MOESM1_ESM.docx]

**Supplementary Information for**

**Surface Plasmon Interference Nanogratings: Wafer-Scale Laser Direct Structuring in Seconds**

Jiao Geng^1,2^, Wei Yan^1,2^, Liping Shi^1,2,*^, and Min Qiu^1,2,*^

^1^ Key Laboratory of 3D Micro/Nano Fabrication and Characterization of Zhejiang Province, School of Engineering, Westlake University, 18 Shilongshan Road, Hangzhou 310024, Zhejiang Province, China.

^2^ Institute of Advanced Technology, Westlake Institute for Advanced Study, 18 Shilongshan Road, Hangzhou 310024, Zhejiang Province, China.

*shiliping@westlake.edu.cn

[*qiumin@westlake.edu.cn](mailto:*qiumin@westlake.edu.cn)

1. **Supplementary information for the experimental parts**
   1. **Robustness of slit-launched SPPs against surface debris**

When there are several neighbouring silica nanoparticles depositing on the Si film, each of them will independently scatter the incident light and excite SPPs. The interference among them results in a distorted pattern (Fig. S1a), although its orientation is still perpendicular to the laser polarization direction (E*_0_*). However, when there exists an air slit in the Si film, it will overcome the influence from the debris, and leads to a regular parallel standing wave that is parallel to the slit orientation (Fig. S1b).


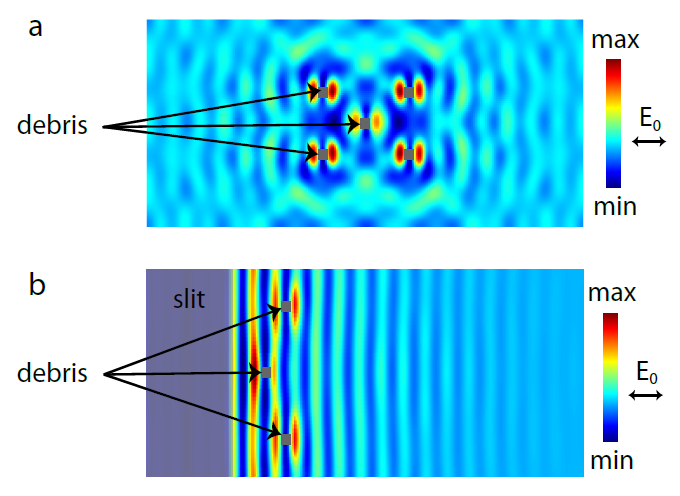


**Figure S1. Schematic illustration of the robustness of slit-induced SPPs against the ablative surface debris by FDTD-based numerical simulation.** Electric field distribution at Si-air interface when existing several debris (a), or an air slit along with several debris (b) depositing on a Si-on-Ag film. The thickness of Si and Ag are 50 nm and 100 nm, respectively. The debris are assumed to be silica nanoparticles, with a size of 400 nm × 400 nm × 50 nm. The illuminated source is a plane at 1030 nm and polarizes along *x*-direction.

- 1. **Material analysis of laser-induced oxidative nanogratings**

To investigate the chemical components of the produced nanogratings (Fig. S2a), we perform EDX (Fig. S2b-S2d) and XPS (Fig. S2e-S2f) analysis. The two-dimensional element maps confirm that there exists periodic distribution of oxygen accumulated in the nanogratings, forming numerous SiO2 and small amount of SiO on the sample surface (Fig. S2e). Using Ar^+^ to etch the sample and investigate the depth profile of the nanogratings, we find that the amount of oxidized silicon, that is, the degree of oxidation, exponential decreases over depth (Fig. S2f).


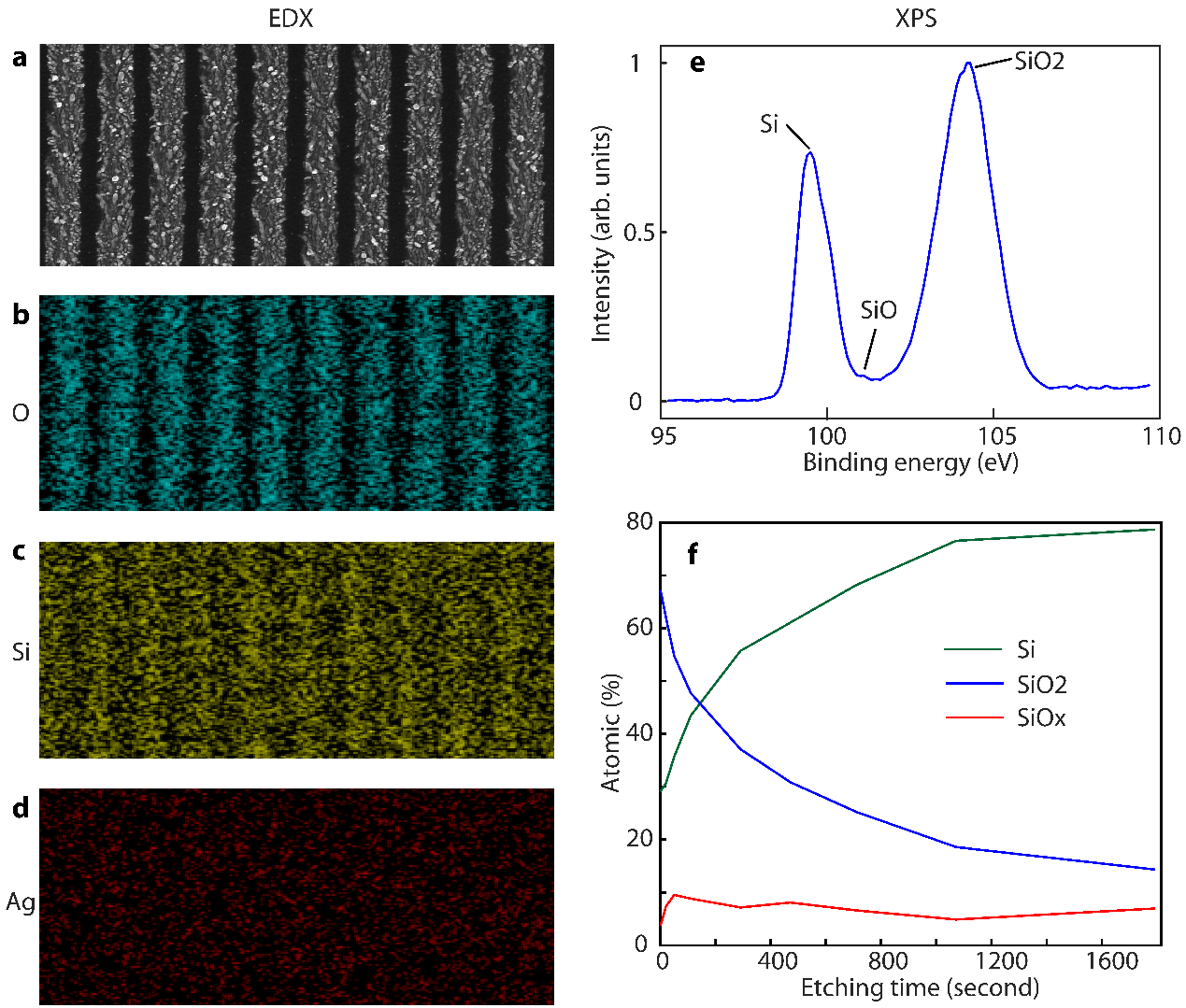


**Figure S2. EDX and XPS analysis of oxidation-induced nanogratings.** (a), SEM image and corresponding two-dimensional EDX maps of elements oxygen (b), silicon (c), and silver (d). The surface XPS spectrum (e), and depth profile (f) of the oxidation nanogratings.

- 1. **Height measurement of the oxidative nanogratings**

We employ a Ga^+^-based focused ion beam to mill the nanogratings and observe their cross-sectional view, as shown in Fig. S3a. It suggests that the oxidation is confined in the Si layer, while the Ag film is unaffected. The oxidation process has penetrated to the interface between Si and silver films. Furthermore, it confirms that the ridges of the nanogratings are composed of numerous nanoparticles. We further measure the height profile of the oxidation-induced nanogratings by an atomic force microscope, as shown in Fig. S3b. The average height is measured to be ~ 50 nm, that is, the thickness of the Si film.


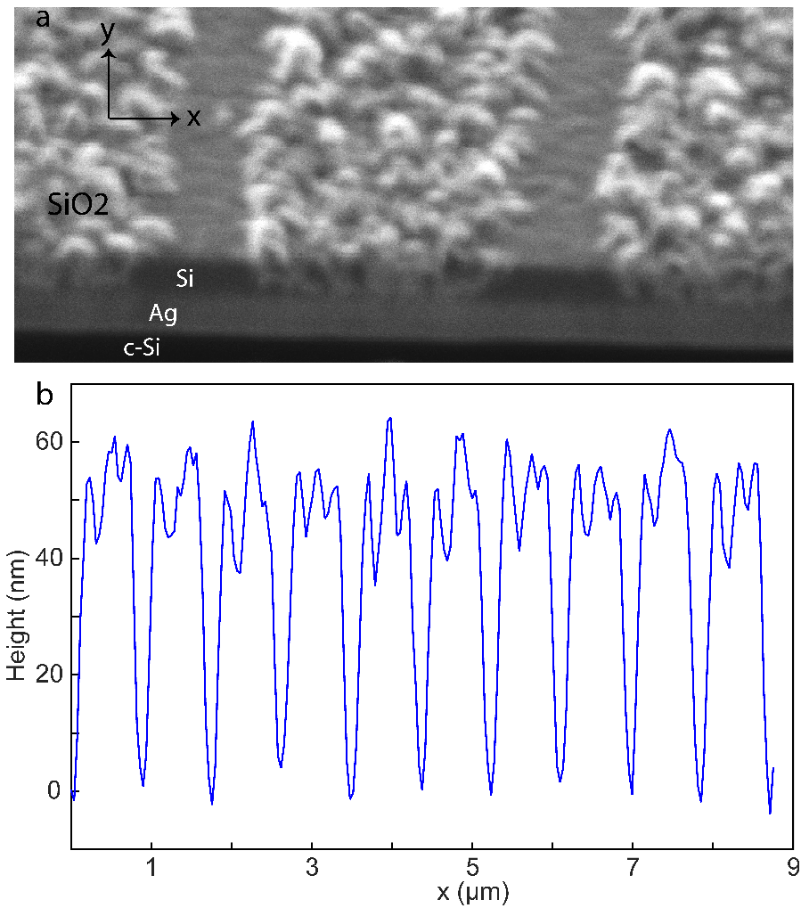


**Figure S3. Cross-sectional view and height profile of the oxidation-induced nanogratings.** (a), The cross-sectional view is milled by a Ga^+^-based focused ion beam and imaged by a He^+^-based focused ion beam. c-Si is the substrate of the Si-on-Ag absorber. (b), the height of oxidation nanogratings that is obtained by atomic force microscopy.

- 1. **Flexibility of the femtosecond laser-wrote nanogratings**

The far-field femtosecond laser-induced self-organization is a flexible technique. For instance, it can produce highly regular nanogratings on a non-planar surface with a curvature radius of 20 cm (Fig. S4). This is due to the insensitivity of the oxidation or ablation nanogratings to variations in laser intensity.


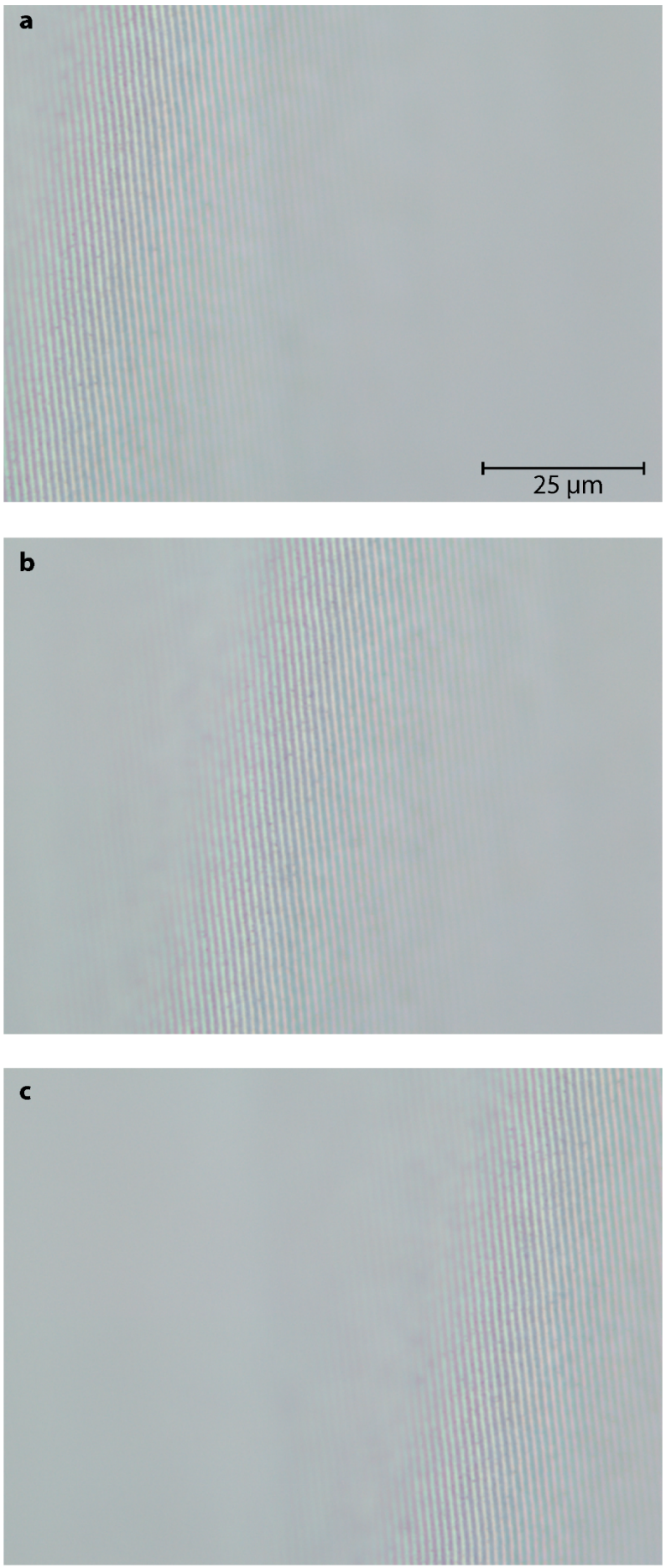


**Figure S4. Manufacturing nanogratings on curved surfaces.** (a-c) Optical microscopy images on different imaging planes, showing nanogratings fabricated on a concave mirror with a curvature radius of 20 cm.

We can also fabricate two-dimensional gratings by scanning with a linear polarization, followed by a second scanning with an orthogonal polarization (Fig. S5a), or directly by scanning with a circularly polarized laser (Fig. S5b).


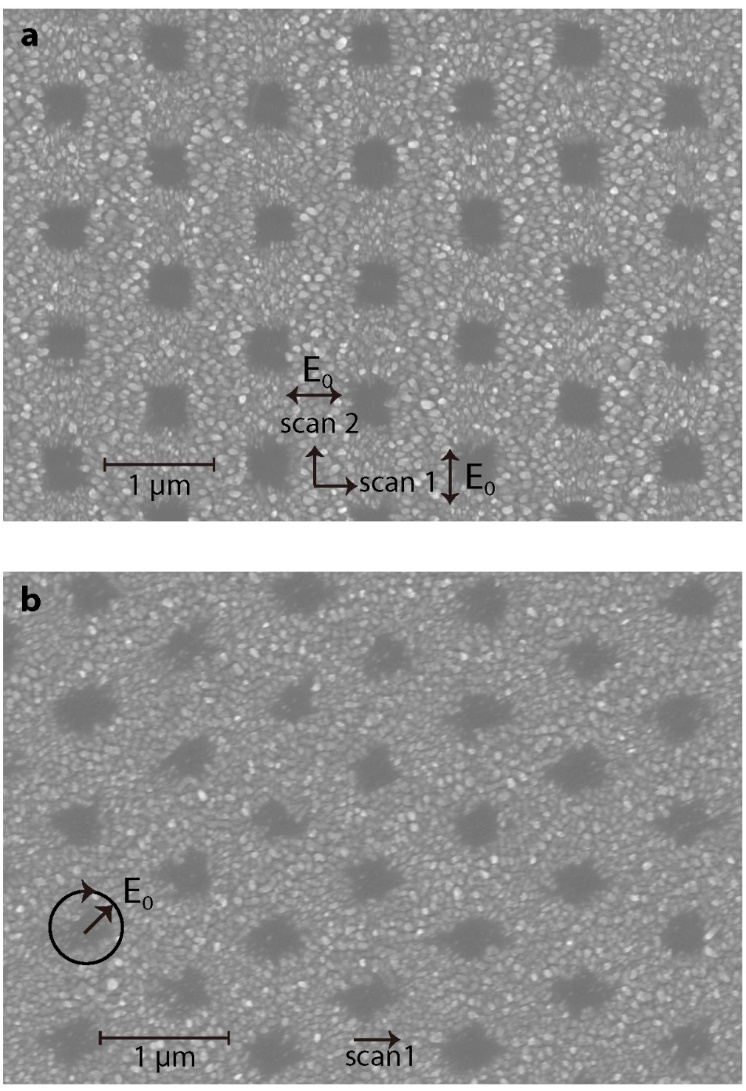

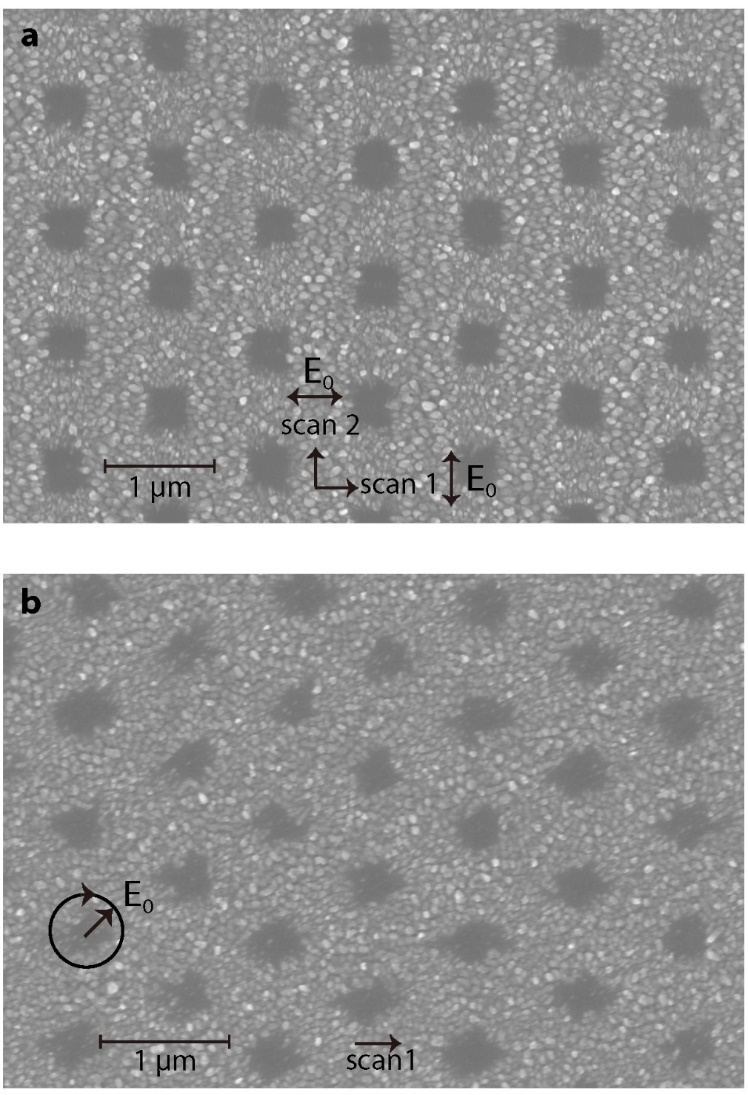


**Figure S5. Two-dimensional nanogratings.** SEM images of two-dimensional gratings that are fabricated by (a), two-step scans of the beams with orthogonal polarizations and (b), single-step scan with circularly polarized femtosecond laser.

When thinning the Si film from 50 to 30 nm, the resonant wavelength shifts from 1000 to 700 nm (Fig. S6a). Correspondingly, the colour of the film changes from brown to light blue (Fig. S6b). To match the resonant spectra of the thin-film absorber, we tune our laser wavelength by an optical parametric amplification (OPA) system. A highly regular nanograting with periodicity of 640 nm is produced by a *λ* = 700 nm laser (Fig. S6c). Our approach is universal to metallic substrate. For instance, when the substrate is Cu, we tune the resonant laser wavelength to *λ* = 750 nm and obtain Si nanogratings with *Λ* = 680 nm (Fig. S6d). At *λ* = 1030, the periodicity of grating is *Λ* = 910 nm. In the spectral range that we can access to (700-1030 nm), we find that he ratio between periodicity and laser wavelength is nearly unchanged, that is, *Λ* / *λ* ≈ 90%.

On-the-fly control over the periodicity should be possible by tunning the laser wavelength, as indicated by the Eq. (1) in the main text. However, the shortest laser wavelength that we can access is *λ* = 700 nm. The ultimate limit of the periodicity depends on the conditions for exciting SPPs, that is, the real part of the metallic permittivity should be negative. Most of metals only support SPPs at visible and near-infrared ranges. Nevertheless, in order to obtain smaller periodicity, one can utilize Al as the substrate metal, because it supports SPPs at ultraviolet spectral range. For example, at *λ* = 200 nm, the permittivity of Al is $\varepsilon_{Al}=-3.6+0.4i$, [1]. The wavelength of SPPs is always shorter than that of incident light. Therefore, nanogratings with sub-200 nm periodicity can be principally achieved.


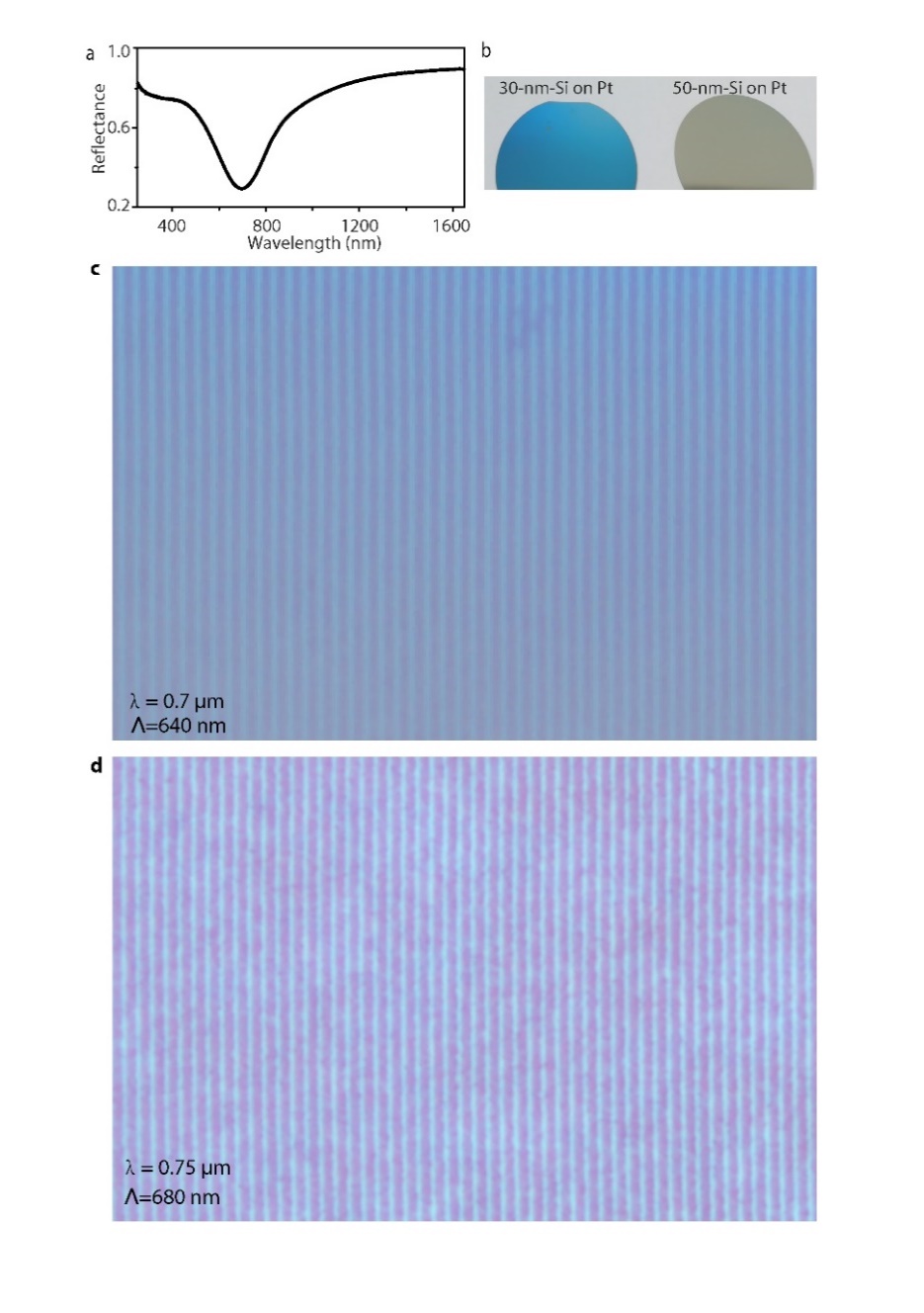


**Figure S6. Nanogratings fabricated on 30-nm-Si coating on metallic film.** (a) normal incidence reflection spectrum of SOM with Si of 30 nm. (b) Photograph of various thickness of Si film deposited on Pt films. The variation of Si thickness can shift the resonant wavelength and thus the colour. (c) Optical microscopy image of nanograting on 30-nm-thick Si supported with 100-nm-thick Pt. The laser wavelength, grating periodicity and scanning speed are 0.7 μm, 0.64 μm and 200 μm s^-1^, respectively. (d) Optical microscopy image of nanograting on 30-nm-thick Si supported with 100-nm-thick Cu. The laser wavelength, grating periodicity and scanning speed are 0.75 μm, 0.68 μm and 200 μm s^-1^, respectively.

- 1. **Irregular nanogratings at intermediate scanning speed**

As shown in Fig. S7, when the scanning speed is near 1 mm s^-1^, the regularity of the gratings is poor. This may be because both oxidation and ablation effects occur in this speed range. A high number of debris, which are most likely to be SiO2 micro/nano particles, randomly deposit on the surface. They strongly disturb the SPPs and thus result in a distorted nanogratings.


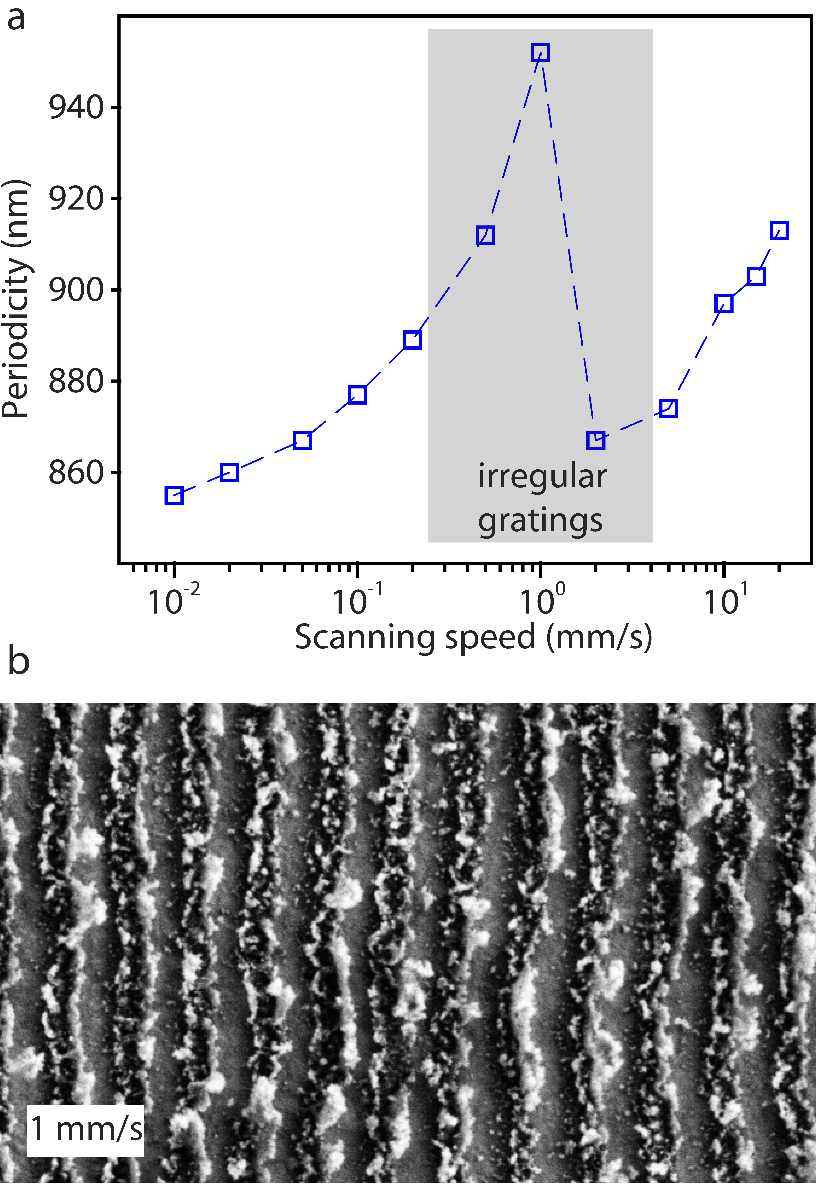


**Figure S7. Irregular Nanogratings** manufactured at scanning speed of 1 mm s^-1^.

- 1. **Material analysis of the ablative nanogratings**

Figure S8 shows the chemical components, height profile analysis of the nanogratings that are produced at high scanning speed, that is 20 mm s^-1^. Unlike the oxidation-dominated nanogratings in Fig. S2, here the periodic distribution of oxygen is not observed (Fig. S8b). Alternatively, we find a periodic distribution of silver (Fig. S8d). This is because the Si coating has been ablated, and thus the silver film has exposed in air. The ablation of Si is directly evidenced by the cross-sectional view of the nanogratings (Fig. S8e), which is cut by a Ga^+^-based focused ion beam is imaged by a high-resolution scanning electron microscope. Accompanied by the ablation process, some nanoparticles redeposit at the Si nanolines, as shown by the SEM images in Fig. S8a and Fig. S8e. Due to the redeposit of these nanoparticles, the height of the ablative nanogratings is increase up to 120 nm, as confirmed by the atomic force microscope (Fig. S8f). Figure S8g-S8h schematically illustrate the difference between the oxidation- and ablation-induced nanogratings. The oxidation nanogratings form oxidation nanolines, with oxygen injecting into the Si layer, while in the ablation case, atoms ejecting from the silicon and some of them redeposit as a recast layer. As a result, the height of the ablative nanograting is higher than its counterpart, as confirmed by the AFM in Fig. S3b and Fig. S8f.


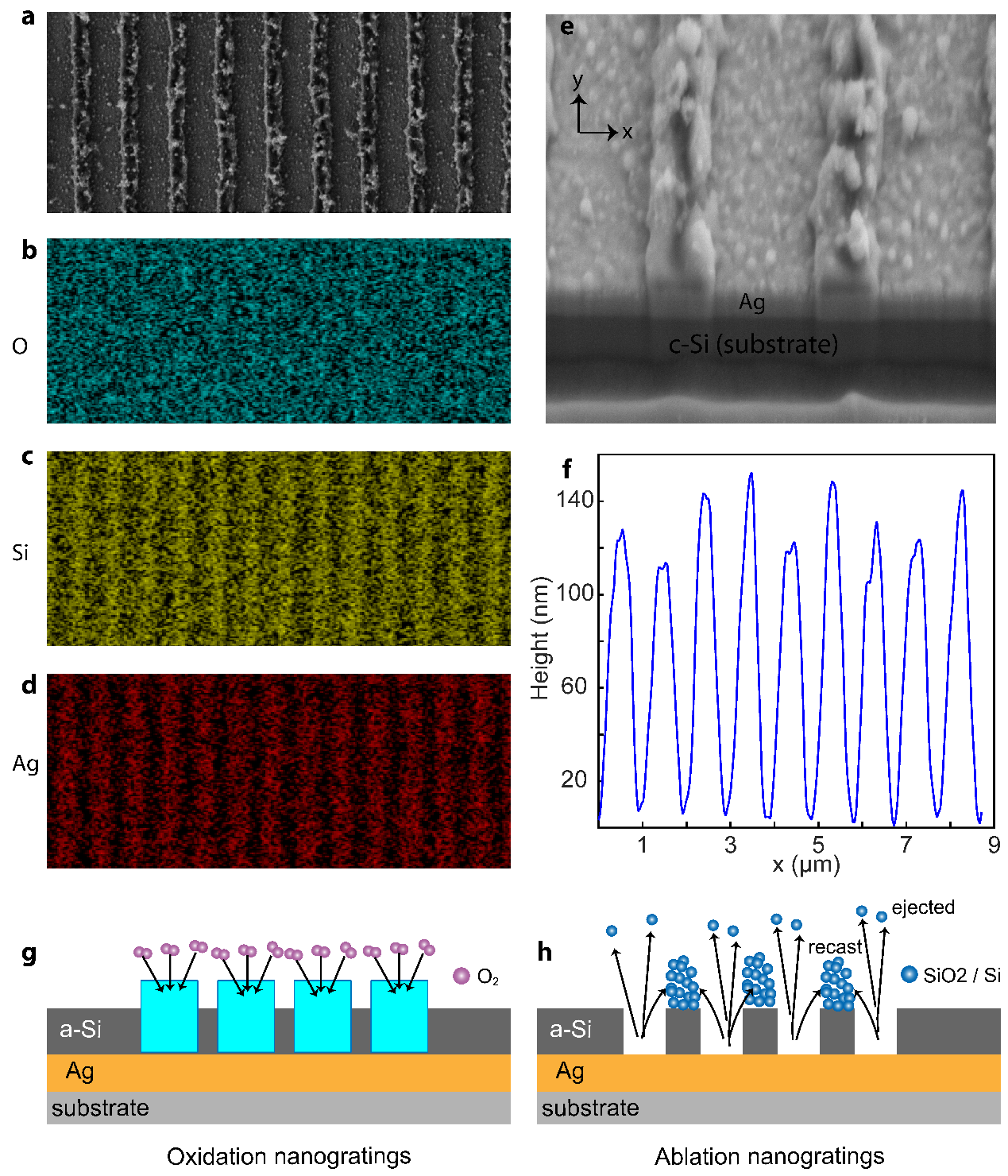


**Figure S8. EDX, FIB, and AFM analysis of ablative nanogratings.** (a), SEM image and corresponding two-dimensional EDX maps of elements oxygen (b), silicon (c), and silver (d) of a nanograting that is fabricated by scanning speed of 20 mm s^-1^. (e), cross-sectional view of the ablative nanograting, which is cut by a Ga^+^-FIB. (f) Height profile of the ablative nanogratings, measured by an atomic force microscope. Schematic illustration of the process of oxidation (g) and ablation (h) nanogratings.

- 1. **Wetting ability of the nanogratings**

The wetting ability of solid surface is related to its intrinsic interaction energy with water and its surface roughness. On the one hand, laser-induced oxidation produces SiOx layer on Si, enhancing its hydrophilicity. [1] On the other hand, the nanogratings increase the surface roughness. According to the Wenzel model,[2] the apparent (macroscopic) contact angle of a water droplet on a rough surface can be expressed as cos(*θ_r_*) = *r* · cos(*θ_f_*), where *θ_r_* is the apparent contact angle, *θ_f_* is the intrinsic contact angle on the flat surfaces, *r* is the surface roughness factor, defined as the ratio between the actual and projected surface areas of the sample. The formation of nanogratings evidently increases the surface roughness, that is, *r* > 1. As the roughness factor increases, wettability changes from hydrophobic to more hydrophobic or from hydrophilic to more hydrophilic. The both changes surface composition and the roughness result in a superhydrophilicity of the nanogratings (Fig.S9a-S9c). The contact angle of the pristine film is 40°, while reaching to <5° on the nanogratings with a spreading time of 0.52 sec.

Superhydrophilicity is attracting interest because of its potential in water harvesting, microfluidics, and self-cleaning applications. Compared with the conventional techniques such as thermal embossing or chemical etching, controllable fabrication of wetting surface by ultrafast lasers has been confirmed as a more suitable industrial technique. [3] However, the laser-induced superhydrophilicity is usually not stable in ambient environment, changing to superhydrophobicity within a few weeks. The preservation of superhydrophilicity exceeding 5 weeks can be considered as a durable wetting surface.[3] We find that the superhydrophilicity of ablative nanogratings on our SOM films is rather durable, as shown in Fig. S9d. The reasons of durability are unclear but may be attributed to the deposition of silica nanoparticles.


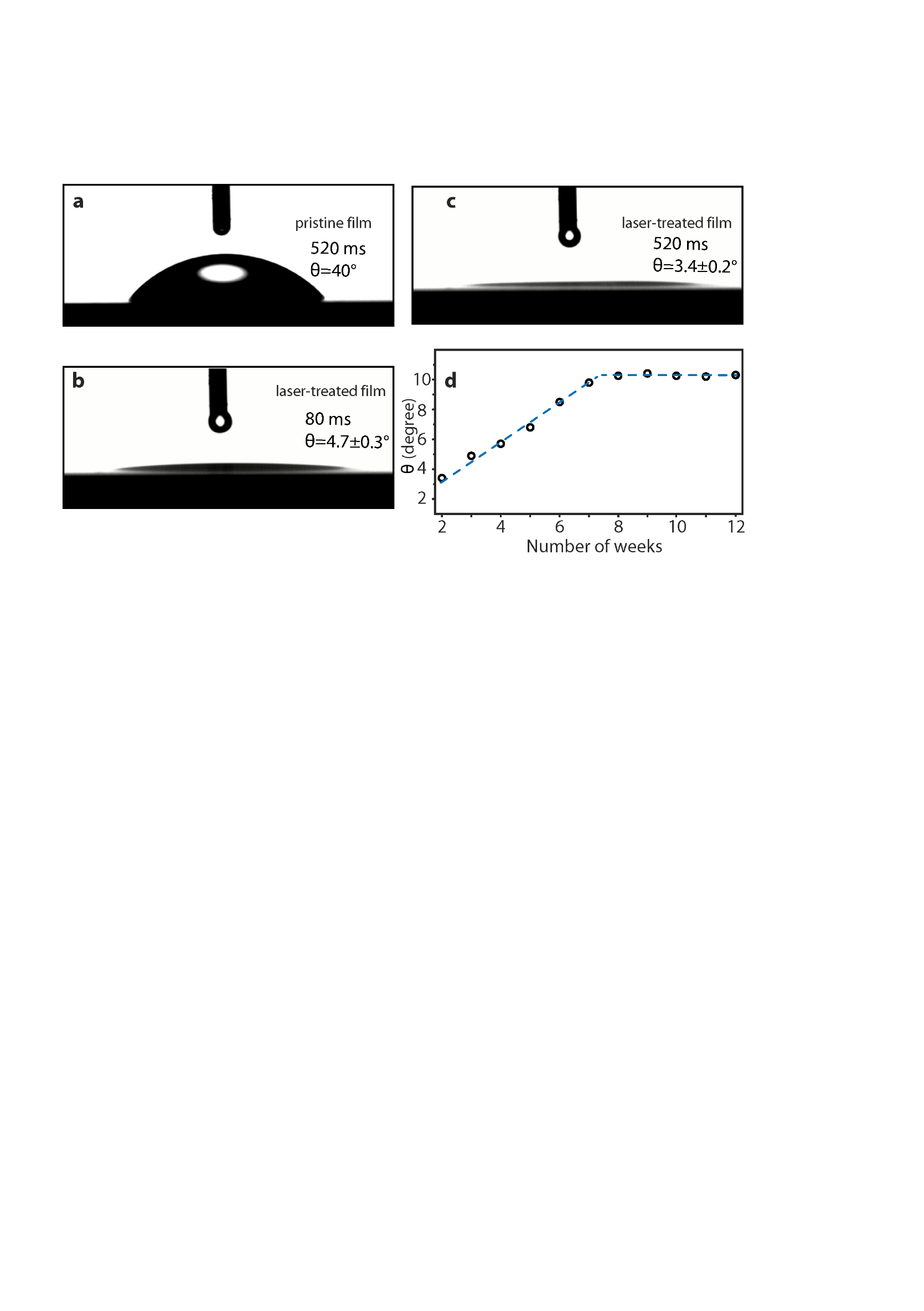


**Figure S9. Hydrophilic property of the laser-induced nanogratings.** contact angle (θ) of a water droplet on the pristine Si-on-Ag film(a), and θ evolution (b, c) on nanogratings. The sample was exposed in ambient for two weeks. (d) θ at spreading time of 520 ms after exposing in air for various number of weeks.

**2. Supplementary information for theoretical parts**

**2.1 Primary nonlinear effects**

The use of intense ultrashort laser pulses leads to noticeable optical nonlinear effects, which render material permittivity of both a-Si films and metal substrates dependences on light density. In this regard, three primary nonlinear effects have to be considered:

1. Excitations of free *e-h* pairs in a-Si, which leads to the Drude permittivity correction [4];
2. Kerr effect and two photon absorptions in a-Si [4];
3. Third-order nonlinearity in metals with complex contributions from intraband electrons (such as ponderomotive nonlinearity), interband electrons and hot electrons [5,6].

The first two considerations, including the excitations of *e-h* pairs, the Kerr nonlinearity and the two-photon absorptions, modify the a-Si to:

$\varepsilon_{a-Si}^{\mathrm{NL}}=\varepsilon_{a-Si}^{L}+\frac{3}{4}\chi_{a-Si}^{(3)} \left| \mathbf{E} \right|^{2}+\varepsilon_{a-Si}^{\mathrm{Drude}}$ (S1)

Here $\varepsilon_{a-si}^{L}$ is the linear permittivity of a-Si. $\chi_{a-Si}^{(3)}$ is the complex-valued nonlinear susceptibility with its real and imaginary parts accounting for the Kerr effect and the two-photon absorptions, respectively. $\varepsilon_{a-Si}^{\mathrm{Drude}}$ characterizes the Drude contribution from the excited *e-h* pairs, which is given by

$\varepsilon_{a-Si}^{\mathrm{Drude}}=-\frac{N_{eh}e^{2}}{\varepsilon_{0}m_{\mathrm{opt}}^{*}(\omega^{2}+\frac{i\omega}{\tau_{D}})}$ (S2)

where $N_{\mathrm{eh}}$ is the density of the excited *e-h* pairs; $\tau_{D}$ denotes the Drude damping time, which is typically in the order of fs [7]; $m_{\mathrm{opt}}^{*}\equiv m_{e}^{*}m_{h}^{*}/(m_{e}^{*}+m_{e}^{*})$ denotes the optical effective mass of a *e-h* pair with $m_{e}^{*}$ and $m_{h}^{*}$ the mobility effective masses of electrons and holes [7]; note that both $\tau_{D}$ and $m_{\mathrm{opt}}^{*}$ depends on $N_{\mathrm{eh}}$ [8].

The density of the excited *e-h* pairs, $N_{\mathrm{eh}}$, can be determined from the following equation that takes the linear and two-photon absorptions into consideration:

$\frac{\partial}{\partial t}N_{\mathrm{eh}}\left( \boldsymbol{r},t \right)=\mathrm{Im}\left( \varepsilon_{a-Si}^{L}+\frac{3}{4}\chi_{a-Si}^{(3)} \left| \mathbf{E} \right|^{2} \right)\frac{\left| \mathbf{E}\left( \boldsymbol{r},t \right) \right|^{2}}{4\hbar}$ (S3)

Note that, in Eq. (S3), the intraband absorption in the Drude term is excluded because it does not excite *e-h* pairs physically. Moreover, the recombination and diffusion of carriers are neglected, and this is justified during excitation [7], where the optical nonlinear effects are important.

Similarly, the metal permittivity modified by the third-order nonlinear effects is given by

$\varepsilon_{m}^{\mathrm{NL}}=\varepsilon_{m}^{L}+\frac{3}{4}\chi_{m}^{(3)} \left| \mathbf{E} \right|^{2}$ (S4)

where $\varepsilon_{m}^{L}$ is the linear permittivity of the metal, and $\chi_{m}^{(3)}$ denotes the nonlinear susceptibility coefficient of the metal.

- 1. **Material parameters**

Equations (S1)-(S4) characterize the primary nonlinear effects. The involved material parameters are specified as follows:

- The linear permittivity of amorphous Si, $\varepsilon_{a-Si}^{L}$, is measured by ellipsometry, which is about $\varepsilon_{a-Si}^{L}\simeq$14.8570+0.2105i at 1.03 $\mu$m (corresponding to refractive index *n* = 3.85 and extinction coefficient *k* = 0.027). The corresponding linear absorption coefficient is evaluated to be *α=4πk/λ*~3300 cm^-1^. The measured extinction coefficient of our Si film is much higher than that of crystalline silicon [9]. This difference can be attributed to defects in deposited amorphous Si films.  In amorphous Si, atoms form a random network and have numerous dangling bonds that cause high absorption loss of near infrared light [10]. The linear permittivity of the metals is taken from Ref. [11].
- $\chi_{a-Si}^{\left( 3 \right)}=\left( 2.21+i1.54 \right)\times{10}^{-19} m^{2} V^{-2}$. Note that the real part of $\chi_{a-Si}^{\left( 3 \right)}$ corresponds to the nonlinear refractive index $n_{2}=4.7\times{10}^{-5} \mathrm{cm}^{2} {GW}^{-1}$ [12], while the imaginary part corresponds to the two-photon extinction coefficient $\beta=2.1 \mathrm{cm} {GW}^{-1}$[12], and the absorption coefficient is calculated to be *β × I_0_* = 2cm GW^-1^ × 800 GW cm^-2^=1600 cm^-1^, where *I_0_* is the laser peak intensity. Therefore, the linear absorption and two-photon absorption have comparable coefficient in our case.
- The first-principle simulation results for the optical effective mass of a *e-h* pair $m_{\mathrm{opt}}^{*}$ and the Drude damping time $\tau_{D}$ are taken [8], and their dependencies on $N_{\mathrm{eh}}$ are plotted in Fig. S10.


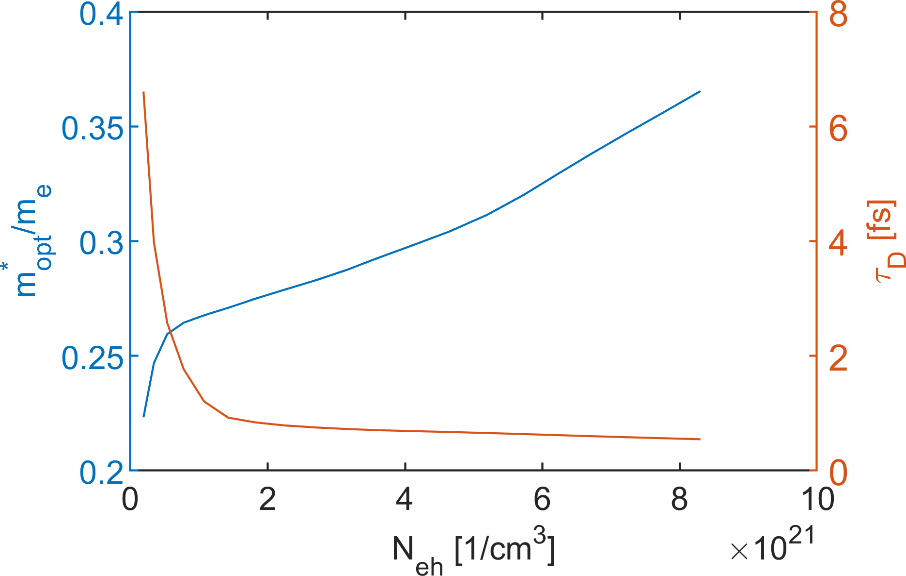


**Figure S10.** Optical effective mass ($m_{\mathrm{opt}}^{*}$) and Drude damping time ($\tau_{D}$) of e-h pairs as functions of e-h pair density $N_{\mathrm{eh}}$, adopted from Ref. [5].

- The third-order nonlinear susceptibilities of metals found varies in the literatures (e.g., see Table 1 in Ref. [6]). Nevertheless, the measured values seem to show consistent values in the order of ${10}^{-18} V^{2}m^{-2}$an ${10}^{-19} V^{2} m^{-2}$around the near-infrared wavelength, agreeing with theoretical predictions, e.g., in Ref. [5]. We here set $\chi_{m}^{(3)}={10}^{-18} m^{2}V^{-2}$.
  1. **Nonlinear Simulations**

To investigate optical responses of silicon-on-metal films (see Fig. S3), we perform numerical simulations by coupling Eqs. (S1) - (S4) with Maxwell’s equations. The electric field of the incident laser pulse is expressed by

$\mathbf{E}_{\mathrm{in}}=\exp\left( -\frac{\left( \frac{z}{c}-t \right)^{2}}{\tau_{p}^{2}} \right)\exp\left( -i\omega_{0}\left( \frac{z}{c}-t \right) \right)\hat{y}$ (S5)

where $\tau_{p}=$65 fs, $\omega_{0}$ is the central frequency of the pulse (corresponding to a light wavelength of 1.03 $\mu$m). The pulse width in the propagation *z* direction is $2\tau_{p}c\simeq40 \mu$m, which is significantly larger than the film thickness (50 nm) plus the skin depth of the metal substrate (tens of nanometers). Therefore, the incident laser pulse can be safely reduced to

$\mathbf{E}_{\mathrm{in}}\simeq\exp\left( -\frac{t^{2}}{\tau_{p}^{2}} \right)\exp\left( -i\omega_{0}\left( \frac{z}{c}+t \right) \right)\hat{y}$ (S6)

where the central z coordinate of the Si-film is set to be $z=0$.

The incident pulse drives the system and excites time-dependent scattered electric fields, $\mathbf{E}_{\mathrm{sca}}$, which is described by Maxwell’s scattering-wave formalism

$\boldsymbol{\nabla}\times\boldsymbol{\nabla}\times\mathbf{E}_{\mathrm{sca}}\boldsymbol{(r},t\boldsymbol{)+}\frac{\varepsilon^{\mathrm{NL}}}{c^{2}} \frac{\partial^{2}}{\partial t^{2}}\mathbf{E}_{\mathrm{sca}}\boldsymbol{(r},t\boldsymbol{)+}\frac{\varepsilon^{\mathrm{NL}}-1}{c^{2}} \frac{\partial^{2}}{\partial t^{2}}\mathbf{E}_{\mathrm{in}}\boldsymbol{(r},t\boldsymbol{)=}0$ (S7)

Further, we express $\boldsymbol{E}_{sca}$ by explicitly separating the fast temporal oscillations at the central frequency from the low envelope variation,

$\mathbf{E}_{\mathrm{sca}}\left( \boldsymbol{r},t \right)\boldsymbol{=}\mathbf{e}_{\mathrm{sca}}\left( \boldsymbol{r},t \right)\exp\left( -i\omega_{0}t \right)$ (S8)

Injecting Eqs. (S6) and (S8) into Eq. (S7), we obtain that

$\boldsymbol{\nabla}\times\boldsymbol{\nabla}\times\mathbf{e}_{\mathrm{sca}}\left( \boldsymbol{r},t \right)\boldsymbol{-}\frac{\omega_{0}^{\boldsymbol{2}}\varepsilon^{\mathrm{NL}}}{c^{2}} \mathbf{e}_{\mathrm{sca}}\left( \boldsymbol{r},t \right)\boldsymbol{+}\frac{\varepsilon^{\mathrm{NL}}}{c^{2}}\frac{\partial^{2}}{\partial t^{2}}\mathbf{e}_{\mathrm{sca}}\left( \boldsymbol{r},t \right)\boldsymbol{+}2\omega_{0}i\frac{\varepsilon^{\mathrm{NL}}}{c^{2}}\frac{\partial}{\partial t}\mathbf{e}_{\mathrm{sca}}\left( \boldsymbol{r},t \right)\boldsymbol{-}\frac{\varepsilon^{\mathrm{NL}}-1}{c^{2}}\left( \omega_{0}^{2}\boldsymbol{+}\frac{4i\omega_{0}t}{\tau_{p}^{2}}\boldsymbol{+}\frac{2}{\tau_{p}^{2}}\boldsymbol{-}\frac{4t^{2}}{\tau_{p}^{4}} \right)\exp\left( -\frac{t^{2}}{\tau_{p}^{2}} \right)\exp\left( -\frac{i\omega_{0}z}{c} \right)=0$

(S9)

The optical responses mainly take place during and shortly after the laser pulse. In this period ($|t|$<$\tau_{p}$, roughly), referring to Eq. (S9), we have $\omega_{0}^{2}\boldsymbol{+}\frac{4i\omega_{0}t}{\tau_{p}^{2}}\boldsymbol{+}\frac{2}{\tau_{p}^{2}}\boldsymbol{-}\frac{4t^{2}}{\tau_{p}^{4}}\boldsymbol{\simeq}\omega_{0}^{2}$, and, moreover, we similarly assume that $\left| \frac{\partial^{2}}{\partial t^{2}}\mathbf{e}_{\mathrm{sca}}\left( \boldsymbol{r},t \right) \right|\boldsymbol{\ll}{\omega_{0}^{\boldsymbol{2}}\mathbf{e}}_{\mathrm{sca}}\left( \boldsymbol{r},t \right)$ and $2\omega_{0}i\frac{\partial}{\partial t}\mathbf{e}_{\mathrm{sca}}\left( \boldsymbol{r},t \right)\boldsymbol{\ll}{\omega_{0}^{\boldsymbol{2}}\mathbf{e}}_{\mathrm{sca}}\left( \boldsymbol{r},t \right)$. Consequently, Eq. (S9) can be further approximated to

$\boldsymbol{\nabla}\times\boldsymbol{\nabla}\times\mathbf{e}_{\mathrm{sca}}\left( \boldsymbol{r},t \right)\boldsymbol{-}\frac{\omega_{0}^{\boldsymbol{2}}\varepsilon^{\mathrm{NL}}}{c^{2}} \mathbf{e}_{\mathrm{sca}}\left( \boldsymbol{r},t \right)\boldsymbol{-}\frac{\omega_{0}^{\boldsymbol{2}}(\varepsilon^{\mathrm{NL}}-1)}{c^{2}}\exp\left( -\frac{t^{2}}{\tau_{p}^{2}} \right)\exp\left( -\frac{i\omega_{0}z}{c} \right)=0$ (S10)

Equation (S10) is stationary Maxwell’s wave equation at frequency $\omega_{0}$, which, coupling with Eqs. (S1)-(S4), predicts the nonlinear responses and is solved with COMSOL Multiphysics linked with Matlab [13].

- 1. **Simulation Results**

We numerically validate that the excitations of *e-h* pairs in a-Si are the dominant nonlinear effect that impact optical responses, as shown in Fig. S10a. We consider a laser pulse with 130-fs duration and 80 mJcm^-2^ fluence impinges on a silicon-on-Ag thin film with 50-nm thickness. The nonlinear permittivity of a-Si is evaluated at the moment when the pulse peak arrives at the a-Si film. The nonlinear permittivity contributed from the Kerr effects and the two-photon absorptions, $\varepsilon_{a-Si}^{K-TPA}\equiv\frac{3}{4}\chi_{a-Si}^{(3)} \left| \mathbf{E} \right|^{2}$ [cf. Eq. (S1)], and the Drude permittivity due to the excited e *e-h* pairs, $\varepsilon_{a-Si}^{\mathrm{Drude}}$, are plotted along the film thickness direction in Fig. S10b. Apparently, the magnitude of $\varepsilon_{a-Si}^{\mathrm{Drude}}$ is much larger than $\varepsilon_{a-Si}^{K-TPA}$. Moreover, we also plot the nonlinear correction to the Ag permittivity, $\varepsilon_{m}^{(3)}\equiv\frac{3}{4}\chi_{m}^{(3)} \left| \mathbf{E} \right|^{2}$ [cf. Eq. (S2)], which show negligible values.


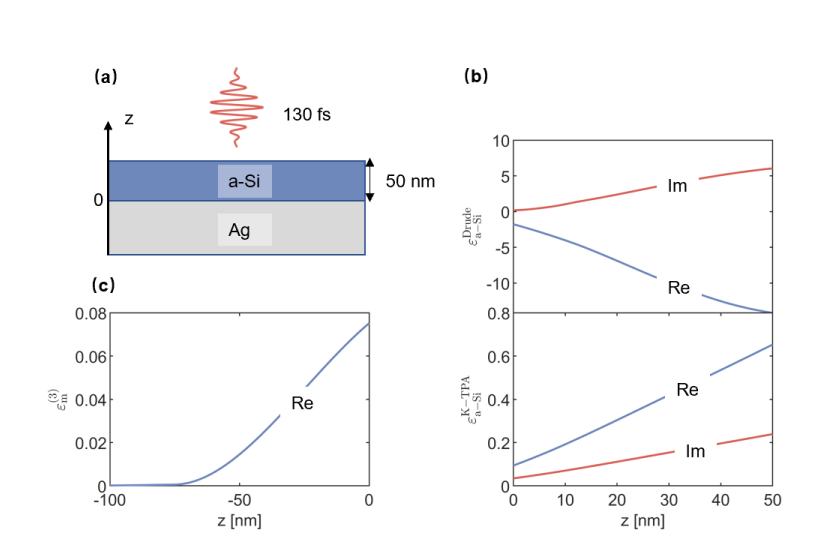


**Figure S11. Numerical comparisons of different optical nonlinear effects in Si-on-Ag thin films.** (a) Sketch of a laser pulse impinging on a Si-on-Ag thin film. The laser pulse has a duration of 130 fs and a fluence of 200 mJ cm^-2^. The a-Si film thickness is 50 nm. **(b-c)** Distributions of the nonlinear corrected permittivity of the a-Si film (**b**) and the Ag substrate (**c**) along the thickness direction of the film at the moment when the pulse peak arrives at the a-Si film. In (**b**), the Drude permittivity due to *e-*h pairs, $\varepsilon_{a-Si}^{\mathrm{Drude}}$, and the contribution from the Kerr effects and the two-photon absorption, $\varepsilon_{a-Si}^{K-TPA}$, are plotted separately in the upper and lower panels, respectively.

We then examine the effects of the pulse fluence on the density of the excited *e-h* pairs and also on the wavelength of the supported SPPs. We compute the evolution of the excited *e-h* pairs in a Si-on-Ag thin film irradiated by a laser pulse with different fluence values, $F=20, 60, 100$ mJ cm^-2^. The pulse parameters and the film thickness are the same as in Fig. S2. Figure S11a plots the saturated density of the excited *e-h* density, $N_{\mathrm{eh}}^{\mathrm{sat}}$, as a function of the laser fluence $F$. It shows that, as $F$ increases, the increasing rate of $N_{\mathrm{eh}}^{\mathrm{sat}}$ gradually declines. This is due to that the fields become less confined in the a-Si film as the increment of the laser fluence that reduces the silicon permittivity (see Fig. 3b). Under the saturated *e-h* pairs, the silicon permittivity is significantly modified, thereby modifying the SPP wavelength, see Fig. S11b. Apparently, as the increment of laser fluences, the SPP wavelength increases, and approaches close to the grating periodicity observed in the experiments (dashed line).


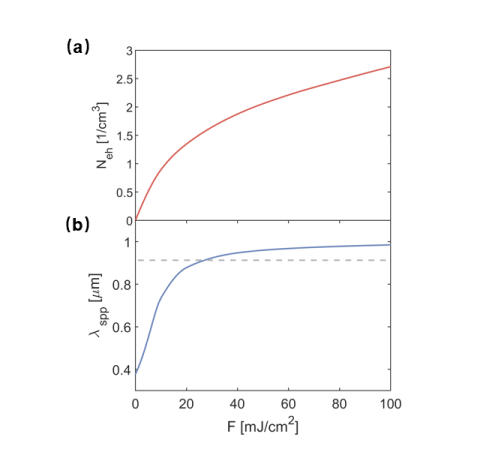


**Figure S12. Density of e-h pairs and SPP wavelength as functions of pulse fluence.** (**b**) Saturated e-h pair density in a Si-on-Ag film as a function of laser fluence. (**c**) SPP wavelength under saturated e-h pairs as a function of laser fluence. The pulse duration and the film thickness are the same parameters as in Fig. S2.

1. **Formation Dynamics of Oxidative and Ablative Nanogratings**

In experiments, oxidative-ridge and ablative-groove nanogratings are formed by exploiting laser scanning at low and high speeds, respectively. The underlying physical mechanisms can be interpreted by noticing the following facts (see the main text for detailed interpretations):

1. Silicon oxidation (SiO_2_) owns a larger ablative threshold than Si; their ablation thresholds are set to be $F_{\mathrm{SiO}2}^{\mathrm{abl}}=2 \mathrm{mJ}\mathrm{cm}^{-2}$ [14] and $F_{a-Si}^{\mathrm{abl}}=0.2 \mathrm{mJ}\mathrm{cm}^{-2}$ [15].
2. Threshold laser intensity to oxidize Si is lower than ablation threshold [16,17], which is set to be $F_{a-Si}^{\mathrm{ox}}=0.1 \mathrm{mJ}\mathrm{cm}^{-2}$, two times smaller than $F_{a-Si}^{\mathrm{abl}}$.
3. The finishing of the oxidation process of Si takes much more laser pulses than of the ablation process, which is straightly deduced from the experiments. More specifically, we observe that the formation of the oxidative-ridge gratings demands the scanning speed lower than 0.5-2 mm s^-1^, which corresponds to an effective pulse number per beam spot area [18] about $\frac{w_{\mathrm{pulse}}f_{\mathrm{rep}}}{v_{\mathrm{scan}}}\simeq20\left[ \mu m \right]\times\frac{20\left[ \mathrm{kHz} \right]}{v_{\mathrm{scan}}}\simeq200\sim800$. Hence, the finishing of the oxidation process uses about a few hundred laser pulses. On the other hand, the ablative gratings are very well produced even when the scanning speed increases 20 mm s^-1^, the maximum value of our experimental setup, implying that the ablation process is rapidly completed using laser pulses less than 10.

Below, we detail how to employ these facts to simulate the formation dynamics of the gratings at different scanning speeds. **Note that the following equations are formulated in the sprit of modelling the essential physics qualitatively instead of precise predictions.** For simplicity, we consider that the scanning beam has a line shape with an infinite length in the *y* direction (12 mm in the experiments) and a width of 20 $\mu$m in the *x* direction (same as the experiments). Next, for this 1D problem, we introduce two functions, $S_{n}^{\mathrm{abl}}(x)$ and $S_{n}^{\mathrm{ox}}$(x) to quantify the ablation and oxidation degrees after irradiations of $n$ pulses.

- $S_{n}^{\mathrm{abl}}$ denotes the ablation ratio of the a-Si film; $S_{n}^{\mathrm{abl}}=$1 and 0 indicates full and null ablations, respectively.
- $S_{n}^{\mathrm{ox}}$ denotes the oxidation ratio of the a-Si film; similarly, $S_{n}^{\mathrm{ox}}=$1 and 0 indicates full and null oxidations, respectively.
- Initially, for $n=0$, we set $S_{0}^{\mathrm{abl}}=0$ and $S_{0}^{\mathrm{ox}}=0$.

Therefore, after *n* pulses, the film is mixed with Si and SiO_2_. The ratio of the remaining Si, denoted by $S_{n}^{\mathrm{Si}}\left( x \right)$, is given by

$S_{n}^{\mathrm{Si}}\left( x \right)\equiv1-S_{n}^{\mathrm{ox}}\left( x \right)-S_{n}^{\mathrm{abl}}\left( x \right)$ (S11)

The existence of SiO_2_ protects the film from ablation. We characterize this effect by setting that the ablation threshold of the film, denoted by $F_{a-Si \& \mathrm{SiO}_{2}}^{\mathrm{abl}}$, is simply the average of Si and SiO_2_, i.e.,

$F_{a-Si \& \mathrm{SiO}_{2}}^{\mathrm{abl}}\equiv\frac{F_{a-Si}^{\mathrm{abl}} S_{n}^{\mathrm{Si}}\left( x \right)+F_{\mathrm{SiO}_{2}}^{\mathrm{abl}} S_{n}^{\mathrm{ox}}\left( x \right)}{S_{n}^{\mathrm{Si}}\left( x \right)+ S_{n}^{\mathrm{ox}}\left( x \right)}$ (S12)

Knowing $S_{n}^{\mathrm{abl}}(x)$ and $S_{n}^{\mathrm{ox}}$(x), we define the following dynamic rules to update $S_{n+1}^{\mathrm{abl}}(x)$ and $S_{n+1}^{\mathrm{ox}}$(x):

$S_{n+1}^{\mathrm{abl}}\left( x \right)=S_{n}^{\mathrm{abl}}\left( x \right)$ and $S_{n+1}^{\mathrm{ox}}\left( x \right)=S_{n}^{\mathrm{ox}}\left( x \right)$,

if $I_{n+1}\left( x \right)<F_{a-Si}^{\mathrm{ox}}$ (S13)

$S_{n+1}^{\mathrm{abl}}\left( x \right)=S_{n}^{\mathrm{abl}}\left( x \right)$ and $S_{n+1}^{\mathrm{ox}}\left( x \right)=S_{n}^{\mathrm{ox}}\left( x \right)+\frac{1}{N_{\mathrm{ox}}}log(I_{n+1}\left( x \right)/F_{a-Si}^{\mathrm{ox}})$

if $F_{a-Si}^{\mathrm{ox}}<I_{n+1}\left( x \right)<F_{a-Si \& \mathrm{SiO}_{2}}^{\mathrm{abl}}$ and $S_{n}^{\mathrm{ox}}\left( x \right)<1$ (S14)

$S_{n+1}^{\mathrm{abl}}\left( x \right)=S_{n}^{\mathrm{abl}}\left( x \right)+\frac{1}{N_{\mathrm{abl}}}log(I_{n+1}\left( x \right)/F_{a-Si \& \mathrm{SiO}_{2}}^{\mathrm{abl}})$ and

$S_{n+1}^{\mathrm{ox}}\left( x \right)=S_{n}^{\mathrm{ox}}\left( x \right)-\frac{1}{N_{\mathrm{abl}}}log(I_{n+1}\left( x \right)/F_{a-Si \& \mathrm{SiO}_{2}}^{\mathrm{abl}})\frac{S_{n}^{\mathrm{ox}}\left( x \right)}{S_{n}^{\mathrm{Si}}\left( x \right)+ S_{n}^{\mathrm{ox}}\left( x \right)}$

if $I_{n+1}\left( x \right)>F_{a-Si \& \mathrm{SiO}_{2}}^{\mathrm{abl}}$ and $S_{n}^{\mathrm{abl}}\left( x \right)<1$ (S15)

Here, the effective pulse numbers for oxidation and ablation are set to be $N_{\mathrm{ox}}=400$ and $N_{\mathrm{abl}}$=3, respectively, which are qualitatively consistent with our experimental observations as discussed above. The dependencies of the oxidization and ablation rates on the intensity are modelled by log functions [12].


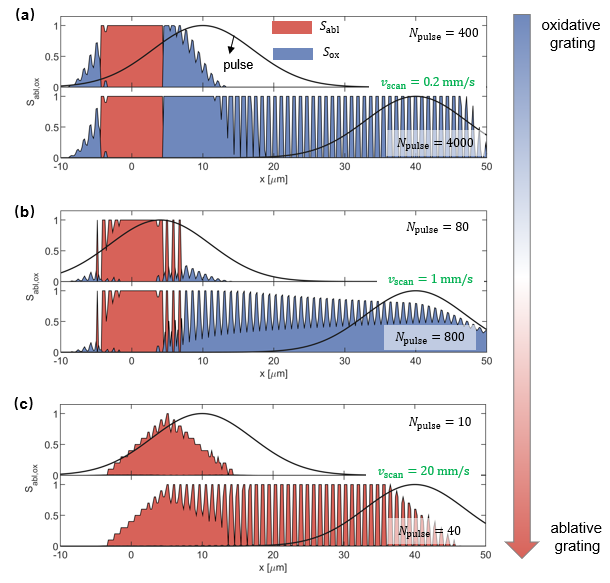


**Figure S13. Formation of oxidative and ablative nanogratings by varying laser-scanning speeds.** The spatial profile of incident line-shaped laser pulses is set to be $E_{\mathrm{in}}\left( x \right)=E_{0}exp(-\left( x-v_{\mathrm{scan}}t \right)^{2}/w_{\mathrm{pulse}}^{2})$ with $v_{\mathrm{scan}}=0.2, 1, 20 mm s^{-1}$ (**a**, **b** and **c**, respectively) and $w_{\mathrm{pulse}}=10\mu m$, and $\left| E_{0} \right|^{2}$ corresponding to a fluence of 0.24 mJ cm^-2^ slightly above the ablation threshold of silicon. At each scanning speed, oxidation and ablation degrees, $S_{n}^{\mathrm{abl}}(x)$ and $S_{n}^{\mathrm{ox}}$(x), are computed by solving Eqs. (S11)-(S13), and illustrated for two different pulse numbers ($N_{\mathrm{pulse}}$).

In Eqs. (S13)-(S16), $I_{n+1}$ is the averaged light intensity in the a-Si film irradiated by the $(n+{1)}^{\mathrm{th}}$pulse. $I_{n+1}=\left| E_{\mathrm{in}}+ E_{n+1}^{\mathrm{spp}} \right|^{2}$, where $E_{\mathrm{in}}$ denotes the incident electric fields, and $E_{n+1}^{\mathrm{spp}}$ denotes the scatted electric fields carried by SPPs. We determine $E_{\mathrm{spp}}$ from the following scattering equation:

$E_{n+1}^{\mathrm{spp}}\left( x \right)=\alpha\int\left[ S_{n}^{\mathrm{abl}}\left( x \right)+S_{n}^{\mathrm{ox}}\left( x \right) \right]\exp\left( ik_{\mathrm{spp}}\left| x-x^{'} \right| \right) \left( E_{\mathrm{in}}\left( x^{'} \right)+E_{n+1}^{\mathrm{spp}}\left( x^{'} \right) \right) dx'$ (S16)

Here $k_{\mathrm{spp}}$ denotes the wavenumber of the SPPs; the phase term characterizes the propagation of SPPs on the film surface; $\alpha$ denotes the excitation factor of the SPPs.

Coupling Eqs. (S13) - (S16), we simulate the formation dynamics of the nanogratings. In Fig. S12, additional numerical results, which are supplemental to Fig. 3g and 3f in the main text, are plotted to illustrated more detailed features of forming oxidative and ablative gratings at low and high scanning speeds, respectively. Note in simulations, referring to Eq. (S16), we set$k_{\mathrm{spp}}=2\pi/\lambda_{\mathrm{spp}}(1+0.05i)$, with $\lambda_{\mathrm{spp}}=0.9 \mu m$ (adopted from the grating periodicity observed in the experiments), and $\alpha=1.95 \mu m^{-1}$ ensuring that the excited SPPs and incident electric fields can interfere with each other adequately.

The simulations in Fig. S13 show that, as the laser pulses impinge on the a-Si film, the ablative grooves firstly form around the central position where the laser starts scanning (i.e., x=0), which well agrees with the experimental observation in Fig. 1c in the main text. Then, as the delivered pulse numbers increase, the regular nanogratings gradually form. Specifically, at a low scanning speed, e.g., $v_{\mathrm{scan}}=0.2 mm s^{-1}$ (a), oxidative gratings are produced, while, at a high scanning speed, e.g., $v_{\mathrm{scan}}=20 mm s^{-1}$ (c), we instead obtain ablative gratings. With an intermediary-speed case, e.g., $v_{\mathrm{scan}}=1 mm s^{-1}$ (b), the grating regularity is obviously degenerated, and the partially oxidized grating is seen.

**References**

[1] Williams R. and Goodman A. M., Appl. Phys. Lett. 25, 531 (1974).

[2] Wenzel, R.N., Resistance of solid surfaces to wetting by water, Ind. Eng. Chem. 28, 988 (1936).

[3] Rajan, R. A. et al. Femtosecond and picosecond laser fabrication for long-term superhydrophilic metal surfaces. Opt. & Laser Technol. 143, 107241 (2021).

[4] Chambonneau, M., Grojo, D., Tokel, O., Ilday, F.Ö., Tzortzakis, S. and Nolte, S., *In-Volume Laser Direct Writing of Silicon—Challenges and Opportunities*, Laser Photonics Rev. **15**, 11 (2021).

[5] Ginzburg, P., Hayat, A., Berkovitch, N., Orenstein, M., *Nonlocal ponderomotive nonlinearity in plasmonics,* Opt. Lett. **35**, 1551 (2010).

[6] Boyd, R. W., Shi, Z. and De Leon, I., *The third-order nonlinear optical susceptibility of gold*, Opt. Commun. **326**, 74–79 (2014).

[7] Sokolowski-Tinten, K. and von der Linde, D., *Generation of dense electron-hole plasmas in silicon*. Phys. Rev. B **61**, 2643–2650 (2000).

[8] Sato, S. A., Shinohara, Y., Otobe, T. and Yabana, K., *Dielectric response of laser-excited silicon at finite electron temperature*. Phys. Rev. B - Condens. Matter Mater. Phys. **90**, 1–8 (2014).

[9] C. Schinke, et al. Uncertainty analysis for the coefficient of band-to-band absorption of crystalline silicon, AIP Advances 5, 067168 (2015).

[10] Takei, R. Amorphous Silicon Photonics. Crystalline and Non-crystalline Solids 2016.

[11] Johnson, P. B. and Christy, R. W., *Optical Constants of the Noble Metals.* Phys. Rev. B **6**, 4370–4379 (1972).

[12] Bristow, A. D., Rotenberg, N. and van Driel, H. M., Two-photon absorption and Kerr coefficients of silicon for 850–2200nm. Appl. Phys. Lett. **90**, 191104 (2007).

[13] The COMSOL module files and the related MATLAB files can be requested by contacting W. Y. ([wyanzju@gmail.com](mailto:wyanzju@gmail.com)).

[14] Lenzner, M. et al., *Femtosecond Optical Breakdown in Dielectrics.* Phys. Rev. Lett. 80, 4076–4079 (1998).

[15] Jeschke, H. O. et al.，L*aser ablation thresholds of silicon for different pulse durations: Theory and experiment.* Appl. Surf. Sci. **197–198**, 839–844 (2002).

[16] Bonse, J., Rosenfeld, A. and Krüger, J., *Femtosecond laser ablation of silicon–modification thresholds and morphology*. J. Appl. Phys. **106**, 104910 (2009).

[17] Bonse, J. and Gräf, S., *Maxwell Meets Marangoni—A Review of Theories on Laser‐Induced Periodic Surface Structures.* Laser Photon. Rev. **14**, 2000215 (2020).

[18] Öktem, B. et al. *Nonlinear laser lithography for indefinitely large-area nanostructuring with femtosecond pulses.* Nat. Photonics **7**, 897–901 (2013).
